# Histone chaperones in Arabidopsis and rice: genome-wide identification, phylogeny, architecture and transcriptional regulation

**DOI:** 10.1186/s12870-015-0414-8

**Published:** 2015-02-12

**Authors:** Amit K Tripathi, Khushwant Singh, Ashwani Pareek, Sneh L Singla-Pareek

**Affiliations:** Plant Molecular Biology Group, International Centre for Genetic Engineering and Biotechnology, Aruna Asaf Ali Marg, New Delhi, 110067 India; Stress Physiology and Molecular Biology Laboratory, School of Life Sciences, Jawaharlal Nehru University, New Delhi, 110067 India

**Keywords:** Nucleosome, Histone chaperones, Rice, Arabidopsis, Phylogeny, Microarray, qRT-PCR, Development, Abiotic stress, Biotic stress

## Abstract

**Background:**

Histone chaperones modulate chromatin architecture and hence play a pivotal role in epigenetic regulation of gene expression. In contrast to their animal and yeast counterparts, not much is known about plant histone chaperones. To gain insights into their functions in plants, we sought to identify histone chaperones from two model plant species and investigated their phylogeny, domain architecture and transcriptional profiles to establish correlation between their expression patterns and potential role in stress physiology and plant development.

**Results:**

Through comprehensive whole genome analyses of Arabidopsis and rice, we identified twenty-two and twenty-five genes encoding histone chaperones in these plants, respectively. These could be classified into seven different families, namely NAP, CAF1, SPT6, ASF1, HIRA, NASP, and FACT. Phylogenetic analyses of histone chaperones from diverse organisms including representative species from each of the major plant groups, yeast and human indicated functional divergence in NAP and CAF1C in plants. For the largest histone chaperone family, NAP, phylogenetic reconstruction suggested the presence of two distinct groups in plants, possibly with differing histone preferences. Further, to comment upon their physiological roles in plants, we analyzed their expression at different developmental stages, across various plant tissues, and under biotic and abiotic stress conditions using pre-existing microarray and qRT-PCR. We found tight transcriptional regulation of some histone chaperone genes during development in both Arabidopsis and rice, suggesting that they may play a role in genetic reprogramming associated with the developmental process. Besides, we found significant differential expression of a few histone chaperones under various biotic and abiotic stresses pointing towards their potential function in stress response.

**Conclusions:**

Taken together, our findings shed light onto the possible evolutionary trajectory of plant histone chaperones and present novel prospects about their physiological roles. Considering that the developmental process and stress response require altered expression of a large array of genes, our results suggest that some plant histone chaperones may serve a regulatory role by controlling the expression of genes associated with these vital processes, possibly via modulating chromatin dynamics at the corresponding genetic loci.

**Electronic supplementary material:**

The online version of this article (doi:10.1186/s12870-015-0414-8) contains supplementary material, which is available to authorized users.

## Background

Eukaryotic nuclear DNA is condensed as chromatin in such a dynamic manner that allows its access for various processes including DNA replication, repair, recombination, and transcription. Chromatin comprises nucleosomal repeats in which each nucleosome is composed of a histone octamer with 146 base pairs of DNA wrapped around it [1]. Two molecules of each of the core histones – H2A, H2B, H3, and H4 together comprise the histone octamer [1]. Cellular processes involving DNA often require transient disruption of nucleosome structure via eviction of histones which requires the action of various nuclear factors [2]. Therefore, in order to maintain the dynamic nature of chromatin, histones must be transported, shuttled or ‘piggy-backed’ into the nucleus, assembled onto DNA as nucleosomes, transiently disassembled, replaced or exchanged [3].

Fundamentally, there are two types of components which define chromatin features: DNA binding factors such as transcription factors which regulate specific gene expression, and histone-associated chromatin factors which possess the capacity to change nucleosome structure and hence alter gene expression [4]. The latter class includes enzymes catalyzing covalent modifications of histones such as histone acetyl transferases (HATs), histone deacetylases (HDACs) and histone methyl transferases; ATP-dependent chromatin remodeling factors; and nucleosome assembly/disassembly factors, also known as histone chaperones [4]. Histone chaperones function to assemble or disassemble chromatin both in replication-coupled as well as replication-independent pathways, without the requirement of ATP [4,5]. Their specific function during chromatin assembly and disassembly is to deposit or evict canonical histones and histone variants. In addition, some histone chaperones such as Nucleosome Assembly Protein 1 (NAP1) are involved in the transport of newly synthesized histones into the nucleus, a prerequisite for their incorporation in nucleosomes [6].

While the nucleosomal organization contributes to the regulation of virtually all the cellular processes operating on DNA [5], not the complete pool of cellular histones is found in association with DNA at any given time. Instead, a soluble reservoir of histones is maintained to address challenges during replication stress conditions [7]. Due to the highly basic nature of histones, their presence in a free state may have detrimental effects on the cell due to non-specific charged interactions and aggregation. Histone chaperones prevent such deleterious effects associated with the presence of free histones, by binding to the non-DNA bound histones [7]. Owing to these activities, histones chaperones aid in controlling histone supply and their incorporation into nucleosomes and thus serve a critical role in fundamental processes of the cell such as DNA replication, DNA repair, recombination, and transcription [5,8-12]. Further, recent studies have suggested that histone chaperones might serve as potent effectors of histone modifications [13]. Thus, histone chaperones are of crucial importance in the maintenance of epigenetic information and genome integrity [14,15].

Histone chaperones constitute quite a diverse group of proteins. They share very little sequence similarity among themselves and the only common feature among them is their acidic nature [4,5]. Histone chaperones generally show preferential binding to a particular class of histones. While most are either H3/H4-specific or H2A/H2B-specific, some bind preferentially to linker histone H1 [5,14]. However, some histone chaperones have been shown to bind to more than one class of histones [16]. Evolutionarily, most of the families of histone chaperones are conserved throughout eukaryotes [4,5,14]. They have been extensively studied in yeast and human and have been classified into various families viz. NPM (Nucleoplasmin/Nucleophosmin), NAP (Nucleosome assembly protein), CAF1 (Chromatin assembly factor I), ASF1 (Anti-silencing factor 1), HIRA (Histone regulatory homolog A), FACT (Facilitates chromatin transcription), NASP (Nuclear Autoantigenic Sperm Protein), and SPT6 (Suppressor of Ty element 6). All but CAF1 complex and FACT complex are single subunit proteins. CAF1 consists of three subunits, CAF1A, CAF1B, and CAF1C in case of humans while CAF1p90, CAF1p60, and CAF1p50 in yeast [4]. The FACT complex consists of two subunits viz. SSRP/Pob3 and SPT16 in both human and yeast [4,17].

The physiological roles of histone chaperones in various organisms and the regulation of pathways operating during nucleosome assembly and disassembly are still not very well understood. Nonetheless, mutations in a few genes encoding histone chaperones have been implicated in causing defects in genome stability and gene expression [15]. In humans, altered expression of some histone chaperones has been linked to cancer and other diseases [15]. In plants, genetic studies for a few histone chaperones have been carried out. For example, it has been shown that the simultaneous loss-of-function mutation in three genes of NAP family (triple mutation) results in hypersensitivity to UV-C radiation in Arabidopsis [18]. Besides, mutant analyses have also revealed that the conserved histone chaperone ASF1 is required for cell proliferation during development in Arabidopsis [19]. Further, publicly available microarray-based expression data has suggested differential expression of some histone chaperones viz. ASF1B, FAS1 and NAP1;3 in a few abiotic stress conditions in Arabidopsis [20]. However, not much is known about the complete pool of histone chaperones in plants and their physiological roles remain to be described. Moreover, the regulatory mechanisms contributing toward nucleosome assembly and disassembly in response to various cellular needs in plants and the functions of various classes of histone chaperones vis-à-vis plant development and responses to various stimuli largely remain enigmatic.

In the present study, we have carried out systematic genome-wide analyses to identify histone chaperones belonging to seven different families in the model plants Arabidopsis and rice. Phylogenetic analyses comprising putative histone chaperones from these two plants besides those from an alga, two basal land plants, a conifer, yeast and human suggested several possibilities about their evolution and possible diversification of function in plants. Besides, we have carried out a comparative analysis of their primary architecture and found unique as well as common sequence elements therein. Further, to gain insights into their potential physiological function in plants, we have studied their expression at different stages of plant development, across various plant tissues, and under biotic and abiotic stresses using public microarray repositories and via qRT-PCR. Our findings suggest interesting links between regulation of gene expression mediated by nucleosome assembly/disassembly and various physiological and developmental aspects of the life cycle of plants, which may serve as a starting point for functional characterization studies for an important class of factors regulating chromatin dynamics – histone chaperones.

## Results

### Genome-wide identification of putative histone chaperones in Arabidopsis and rice

To identify the genes encoding histone chaperones in the genomes of Arabidopsis and rice, we utilized profile HMM (Hidden Markov Model) for representative members of each of the histone chaperone families using their sequences from yeast and human and searched the Arabidopsis and rice protein sequence databases (see Methods). The histone chaperones thus identified have been listed in seven different families (with CAF1 family further divided into three sub-families viz. CAF1A, CAF1B, and CAF1C; and FACT family further classified into SSRP and SPT16 sub-families) as given in Tables 1 and 2. In Arabidopsis, we could identify twenty-two genes coding for thirty-four proteins and in rice we found twenty-five genes encoding thirty-one proteins due to the presence of alternative spliced forms (Tables 1 and 2). In Arabidopsis, we found eleven proteins (including splice variants) each of NAP and CAF1 families, four of SPT6, two each of ASF1, HIRA, NASP, and FACT families (Table 1). In rice, we found eleven proteins (including splice variants) belonging to the NAP family, nine to CAF1, one to SPT6, two to ASF1, one to HIRA, one to NASP, and six to FACT family (Table 2). We did not find any protein related to Nucleophosmin (NPM) in both the plant genomes studied. Further, a BLASTp search against the sequenced genomes of thirty-seven diverse plant species (ranging from algae to monocots) present in PLAZA 3.0 Dicot and PLAZA 3.0 Monocot comparative genomics platforms (see Methods) indicated that these genomes also do not harbor any gene encoding a protein related to NPM. As NPM is also absent in yeast (Additional file 1: Table S1), it appears that animals may have acquired NPM later in their evolution.

**Table 1:** **List of putative histone chaperones identified from Arabidopsis showing their classification, predicted intracellular localization, and other biochemical properties**

**Family**	**Sub-family**	**Gene**	**Previous nomenclature (Gene symbol)**	**Protein**	**Locus id**	**pI/Mw (kDa)**	**Localization**	**NLS/NES**
NAP		*AtNAPL1*	*NAP1;1*	AtNAPL1a	AT4G26110.2	4.33/41.6	Nuclear	+/-
				AtNAPL1b	AT4G26110.1	4.38/42.9	Nuclear	+/-
		*AtNAPL2*	*NAP1;2*	AtNAPL2a	AT2G19480.3	4.27/42.8	Nuclear	+/+
				AtNAPL2b	AT2G19480.2	4.29/42.9	Nuclear	+/+
				AtNAPL2c	AT2G19480.1	4.32/43.5	Nuclear	+/+
		*AtNAPL3*	*NAP1;3*	AtNAPL3	AT5G56950.1	4.42/43.2	Nuclear	+/-
		*AtNAPL4*	*NAP1;4*	AtNAPL4	AT3G13782.1	4.52/36.4	Nuclear	+/+
		*AtNAPL5*	*NRP2*	AtNAPL5	AT1G18800.1	4.17/29.4	Nuclear	-/+
		*AtNAPL6*	*NRP1*	AtNAPL6a	AT1G74560.1	4.22/29.4	Nuclear	-/+
				AtNAPL6b	AT1G74560.2	4.22/29.5	Nuclear	-/+
				AtNAPL6c	AT1G74560.3	5.22/30.6	Cytoplasmic	+/+
CAF1	CAF1A	*AtCAF1AL*	*FAS1*	ATCAF1ALa	AT1G65470.2	5.45/92.3	Nuclear	+/-
				ATCAF1ALb	AT1G65470.1	5.49/93.3	Nuclear	+/-
	CAF1B	*AtCAF1BL*	*FAS2*	ATCAF1BLa	AT5G64630.1	6.35/43.7	Nuclear	-/+
				ATCAF1BLb	AT5G64630.2	5.99/54.1	Nuclear	-/-
				ATCAF1BLc	AT5G64630.3	5.82/47.9	Nuclear	-/-
	CAF1C	*AtCAF1CL1*	*MSI1*	AtCAF1CL1	AT5G58230.1	4.69/48.1	Nuclear	-/+
		*AtCAF1CL2*	*MSI3*	AtCAF1CL2	AT4G35050.1	4.55/47.9	Nuclear	-/-
		*AtCAF1CL3*	*MSI2*	AtCAF1CL3	AT2G16780.1	4.66/46.7	Cytoplasmic	-/-
		*AtCAF1CL4*	*FVE*	AtCAF1CL4	AT2G19520.1	5.81/55.7	Nuclear	-/-
		*AtCAF1CL5*	*NFC5*	AtCAF1CL5	AT4G29730.1	6.12/53.9	Nuclear	-/-
		*AtCAF1CL6*		AtCAF1CL6	AT2G19540.1	4.89/51.4	Nuclear	-/-
SPT6		*AtSPT6L1*	*GTB1*	AtSPT6L1a	AT1G65440.3	5.88/166.3	Nuclear	+/+
			*GTB1*	AtSPT6L1b	AT1G65440.2	5.12/185.8	Nuclear	+/+
			*GTB1*	AtSPT6L1c	AT1G65440.1	5.08/185.0	Nuclear	+/+
		*AtSPT6L2*	*SPT6L*	AtSPT6L2	AT1G63210.1	5.98/138.1	Nuclear	+/-
ASF1		*AtASF1L1*	*ASF1B*	AtASF1L1	AT5G38110.1	4.02/24.7	Nuclear	-/-
		*AtASF1L2*	*SGA1*	AtASF1L2	AT1G66740.1	4.20/22.1	Nuclear	-/-
HIRA		*AtHIRAL*	*HIRA*	AtHIRALa	AT3G44530.2	6.26/114.3	Nuclear	+/+
				AtHIRALb	AT3G44530.1	6.40/116.4	Nuclear	+/+
NASP		*AtNASPL*		AtNASPLa	AT4G37210.1	4.28/52.2	Cytoplasmic	-/+
				AtNASPLb	AT4G37210.2	4.11/40.5	Nuclear	-/+
FACT	SSRP	*AtSSRPL*	*HMG*	AtSSRPL	AT3G28730.1	5.52/71.6	Nuclear	+/+
	SPT16	*AtSPT16L*	*GTFC*	AtSPT16L	AT4G10710.1	5.70/120.5	Nuclear	+/-

The alternative spliced forms have been named by suffixing lower case letters. pI = Isoelectric point (predicted), Mw = Molecular weight, NLS = Nuclear localization signal, NES = Nuclear export signal. ‘+ ’denotes present; ‘-’denotes absent. Note that most of the putative histone chaperones have their predicted pI in the acidic region.

**Table 2:** **List of putative histone chaperones identified from rice showing their classification, predicted intracellular localization, and other biochemical properties**

**Family**	**Sub-family**	**Gene**	**Protein**	**Locus id**	**pI/Mw**	**Localization**	**NLS/NES**
NAP		*OsNAPL1 (Orysa; NAP1;2)*	OsNAPL1	LOC_Os05g46230.1	4.34/41.6	Nuclear	**-/-**
		*OsNAPL2 (Orysa; NAP1;1)*	OsNAPL2a	LOC_Os06g05660.5	4.35/40.5	Nuclear	**-/-**
			OsNAPL2b	LOC_Os06g05660.4	4.31/41.2	Nuclear	**-/-**
			OsNAPL2c	LOC_Os06g05660.3	4.34/42.1	Nuclear	**-/-**
			OsNAPL2d	LOC_Os06g05660.2	4.35/42.3	Nuclear	**-/-**
			OsNAPL2e	LOC_Os06g05660.1	4.33/42.6	Nuclear	**-/-**
		*OsNAPL3 (Orysa; NAP1;3)*	OsNAPL3	LOC_Os01g51450.1	4.28/34.9	Cytoplasmic	**-/-**
		*OsNAPL4*	OsNAPL4	LOC_Os06g40920.1	4.62/46.9	Cytoplasmic	**-/+**
		*OsNAPL5*	OsNAPL5	LOC_Os04g38620.1	4.29/29.9	Nuclear	**+/-**
		*OsNAPL6*	OsNAPL6	LOC_Os02g36710.1	4.21/28.5	Cytoplasmic	**-/+**
		*OsNAPL7*	OsNAPL7	LOC_Os05g14570.1	4.19/17.7	Nuclear	**-/-**
CAF1	CAF1A	*OsCAF1AL1*	OsCAF1AL1	LOC_Os07g17210.1	5.43/83.2	Nuclear	**+/+**
		*OsCAF1AL2*	OsCAF1AL2	LOC_Os01g67100.1	7.09/100.4	Nuclear	**+/+**
	CAF1B	*OsCAF1BL*	OsCAF1BL	LOC_Os08g01680.1	6.38/55.3	Nuclear	**-/+**
	CAF1C	*OsCAF1CL1*	OsCAF1CL1	LOC_Os03g43890.1	4.77/48.3	Nuclear	**-/+**
		*OsCAF1CL2*	OsCAF1CL2	LOC_Os09g36900.1	4.98/44.7	Cytoplasmic	**-/+**
		*OsCAF1CL3*	OsCAF1CL3a	LOC_Os01g51300.2	5.87/50.1	Cytoplasmic	**-/+**
			OsCAF1CL3b	LOC_Os01g51300.1	5.79/51.0	Cytoplasmic	**-/-**
		*OsCAF1CL4*	OsCAF1CL4	LOC_Os11g03990.1	5.18/51.8	Cytoplasmic	**-/-**
		*OsCAF1CL5*	OsCAF1CL5	LOC_Os12g03822.1	5.18/51.7	Nuclear	**-/-**
SPT6		*OsSPT6L*	OsSPT6L	LOC_Os05g41510.1	5.05/184.0	Nuclear	**+/+**
ASF1		*OsASF1L1*	OsASF1L1	LOC_Os05g48030.1	4.24/21.1	Nuclear	**-/+**
		*OsASF1L2*	OsASF1L2	LOC_Os01g49150.1	5.08/33.4	Cytoplasmic	**-/+**
HIRA		*OsHIRAL*	OsHIRAL	LOC_Os09g39420.1	7.10/106.5	Nuclear	**-/+**
NASP		*OsNASPL*	OsNASPL	LOC_Os07g03070.1	4.36/49.9	Nuclear	**+/+**
FACT	SSRP	*OsSSRPL1*	OsSSRPL1a	LOC_Os01g08970.1	5.52/71.3	Nuclear	**+/-**
			OsSSRPL1b	LOC_Os01g08970.2	5.24/59.6	Nuclear	**+/-**
		*OsSSRPL2*	OsSSRPL2	LOC_Os05g08970.1	5.71/71.0	Nuclear	**+/-**
	SPT16	*OsSPT16L1*	OsSPT16L1	LOC_Os04g25550.1	5.41/118.5	Nuclear	**+/+**
		*OsSPT16L2*	OsSPT16L2	LOC_Os12g26030.1	6.38/120.2	Nuclear	**-/-**
		*OsSPT16L3*	OsSPT16L3	LOC_Os08g31240.1	9.21/114.7	Nuclear	**-/-**

The alternative spliced forms have been named by suffixing lower case letters. pI = Isoelectric point (predicted), Mw = Molecular weight (in kDa), NLS = Nuclear localization signal, NES = Nuclear export signal. ‘+ ’denotes present; ‘-’denotes absent. Note that most of the putative histone chaperones have their predicted pI in the acidic region.

The members of each of the families of histone chaperones, thus identified, have been named as: name of the histone chaperone in humans/yeast, followed by ‘L’ (for ‘like’) and a number (only in case of families having multiple genes) based on their HMM score, with the protein having a higher HMM score getting a lower number followed by lower case letters for the spliced forms in decreasing order of the HMM score for the respective splice variants (see Methods). In cases where prior information was available in databases or literature regarding any of the histone chaperones from Arabidopsis or rice, the HMM-based nomenclature as described has been maintained and the existing names have been mentioned in parentheses both in the text and in Tables 1 and 2.

A comparison of the putative members of various families of histone chaperones as found in these two model plants, Arabidopsis and rice, with histone chaperones from other model eukaryotic genomes such as *Saccharomyces cerevisiae* and *Homo sapiens* using annotated proteins from Uniprot database revealed that these two higher plants have either equal or a higher number of members in five of the histone chaperone families (all except NAP and HIRA) as compared to both yeast and human (Tables 1 and 2; and Additional file 1: Table S1). Arabidopsis and rice both have more members as compared to yeast and human in CAF1C subfamily while rice has a higher number of genes in the FACT family. Further, Arabidopsis possesses two genes encoding SPT6 as compared to one each in yeast and human (Table 1 and Additional file 1: Table S1). These observations indicate an expansion of such gene families in the respective plant species.

### Chromosomal distribution of the genes encoding histone chaperones and detection of duplication events

**Figure 1:**
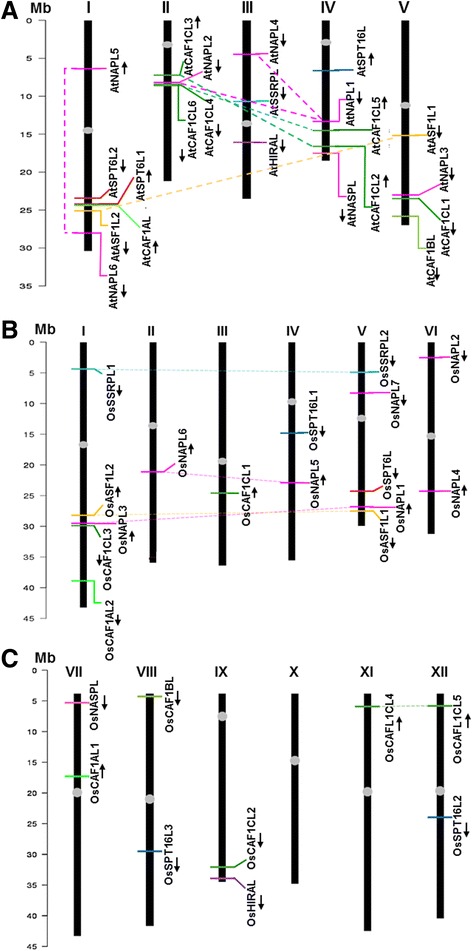
**Chromosomal distribution and segmental duplication events of genes encoding histone chaperones in Arabidopsis and rice.** The karyograms show the chromosomal positions of genes coding for histone chaperones belonging to NAP, CAF1, SPT6, SPT16, SSRP, HIRA, ASF and NASP families/sub-families from **(A)** Arabidopsis and **(B)** rice showing genes located on chromosome 1 to 6, and **(C)** rice showing genes on chromosome 7 to 12. The broken lines connect genes located on duplicated segments of chromosomes with the color of the line representing the color of the histone chaperone family. The chromosomal positions of each of the genes are shown by colored horizontal bars and the orientation of the respective genes has been shown by arrows. Scale is shown at the left (Mb indicates mega base pairs) and the centromeres are represented by oval shapes in gray.

The genes for histone chaperones in Arabidopsis were found to be located across all the five chromosomes, while eleven out of twelve chromosomes of rice possess one or more genes for histone chaperones (Figure 1A,B and C). Interestingly, in Arabidopsis, we found that both the genes of the SPT6 family and three members of the CAF1C sub-family are located in close proximity on chromosome 1 and chromosome 2, respectively (Figure 1A). Further, in rice, one gene each of ASF1 and NAP families and CAF1C sub-family were found to be closely located on chromosome 1 (Figure 1B).

When we addressed as to if one of the reasons for the presence of some multi-membered families of histone chaperones in plants is gene duplication, we found six duplication events in Arabidopsis and five such events in rice. Of these five events in rice, four (two in NAP, and one each in CAF1C and ASF1) were common to those found in Arabidopsis suggesting that these duplication events might have taken place before the divergence of dicots and monocots (Figures 1A,B and C). The other duplication event found in rice is in SSRP family which led to the occurrence of two SSRP genes in rice. *SSRP* is present as a single gene in Arabidopsis (Table 1), human (Additional file 2: Table S2), and several lower plants (Additional file 3: Table S3). Thus, it seems that this duplication event might have led to the expansion of SSRP family in rice. Interestingly, in Arabidopsis, three genes in the NAP family (*AtNAPL1*, *AtNAPL2* and *AtNAPL4*) were found to have arisen from two segmental duplications (Figure 1A). Besides, Arabidopsis has one additional gene in the CAF1C subfamily owing to a duplication event (*AtCAF1CL2*-*AtCAF1CL3*), suggesting it to be an event, taking place in dicots post-divergence of dicots and monocots (Figures 1A-C). Further, segmental duplication might have led to the simultaneous duplication of closely linked genes as found between chromosome 2 and 4 of Arabidopsis (Figure 1A) and 1 and 5 of rice (Figure 1B).

### Phylogenetic analysis of histone chaperones from diverse organisms indicates interesting possibilities about their evolution, histone specificity and function

In order to comment upon the evolutionary relationship amongst members of each of the families and sub-families of histone chaperones from yeast, human, Arabidopsis and rice as well as those from other representative plant species viz. *Chlamydomonas reinhardtii* (a green alga), *Physcomitrella patens* (a bryophyte), *Selaginella moellendorffii* (a pteridophyte), and *Picea abies* (a gymnosperm), phylogenetic trees were constructed. For this purpose, we carried out a similar HMM-based search against the genomes of these four plant species and identified the putative histone chaperones (see Methods). Histone chaperones belonging to the individual family/sub-family were aligned (Additional file 4: Figure S1, Additional file 5: Figure S2, Additional file 6: Figure S3, Additional file 7: Figure S4, Additional file 8: Figure S5, Additional file 9: Figure S6, Additional file 10: Figure S7, Additional file 11: Figure S8, Additional file 12: Figure S9, Additional file 13: Figure S10) and the alignments were subsequently used to generate phylogenetic trees.

**Figure 2:**
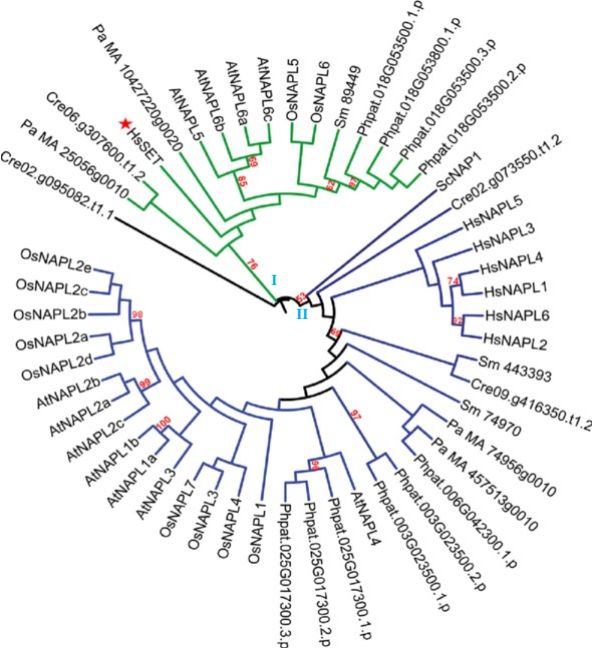
**Phylogenetic reconstruction of NAP family of histone chaperones from various eukaryotic taxa.** A phylogenetic tree was constructed to determine evolutionary distances among the members of NAP family of histone chaperones from *Arabidopsis thaliana* (named by prefixing ‘At’), *Oryza sativa* (named by prefixing ‘Os’), *Chlamydomonas reinhardtii* (represented by the prefix ‘Cre’ with the locus id), *Physcomitrella patens* (represented by the prefix ‘Phpat’ with the locus id), *Selaginella moellendorffii* (named by prefixing ‘Sm’), *Picea abies* (named by prefixing ‘Pa’), *Saccharomyces cerevisiae* (named by prefixing ‘Sc’), and *Homo sapiens* (named by prefixing ‘Hs’). For generation of the tree, the protein sequences were aligned using ClustalX2 and the tree was constructed using MEGA 6.06 and viewed using iTOL. Maximum Likelihood method was used for construction of the tree and the reliability of the branches was inferred from a bootstrap analysis of 1000 replicates. Bootstrap values above 50% have been shown as numbers. Star mark shows the position of HsSET in the tree. The sequence ids of the proteins used for the generation of the tree have been given in Table 1 (for Arabidopsis), Table 2 (for rice), and Additional file 2: Table S2 (for *S. cerevisiae*, and *H. sapiens*) and Additional file 3: Table S3 (for rest of the studied species).

The NAP family was found to be the largest with members from a single species separated into two distinct groups (Figure 2). All NAPs from human (HsNAPs) except HsSET were found to be clustered together in the larger group II. ScNAP1 was also found in the group II although it formed a separate leaf suggesting its lower homology to other members of the group. HsSET clustered in a separate group (Group I) with one protein each from *C. reinhardtii* and *S. moellendorffii* and two proteins (excluding splice variants) each from *P. patens, P. abies,* Arabidopsis and rice*.* HsSET, despite being a member of the NAP family, is known to function quite differently from other NAP family proteins [21,22]. Therefore, our analysis predicts that plant proteins which clustered with HsSET, namely OsNAPL5, OsNAPL6, AtNAPL5, AtNAPL6 and others from the lower plants, might perform functions similar to those of HsSET in the respective plant species. This possibility, however, needs further validation. Other NAP family members from Arabidopsis (except AtNAPL4) and rice were found to cluster together in the group II (Figure 2).

**Figure 3:**
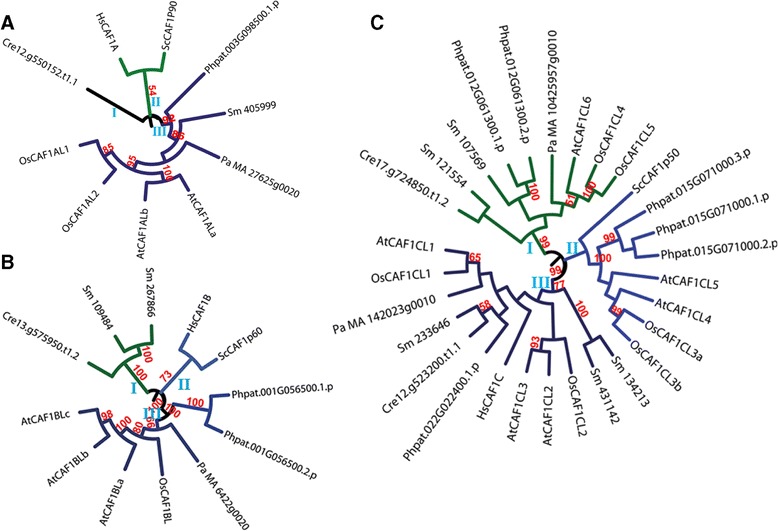
**Phylogenetic analysis of CAF1 family of histone chaperones from diverse eukaryotic taxa.** Phylogenetic trees constructed to determine evolutionary distances among the members of each of the three sub-families of CAF1 viz. CAF1A **(A)**, CAF1B **(B)**, and CAF1C **(C)** from *Arabidopsis thaliana* (named by prefixing ‘At’), *Oryza sativa* (named by prefixing ‘Os’), *Chlamydomonas reinhardtii* (represented by the prefix ‘Cre’ with the locus id), *Physcomitrella patens* (represented by the prefix ‘Phpat’ with the locus id), *Selaginella moellendorffii* (named by prefixing ‘Sm’), *Picea abies* (named by prefixing ‘Pa’), *Saccharomyces cerevisiae* (named by prefixing ‘Sc’), and *Homo sapiens* (named by prefixing ‘Hs’). The protein sequences were aligned using ClustalX2 and the tree was constructed using MEGA 6.06 using Maximum Likelihood method and viewed using iTOL. The reliability of the branches was estimated using bootstrap analyses of 1000 replicates and bootstrap values above 50% have been shown as numbers. Roman numerals show arbitrary numbers given to the phylogenetic groups. Sequence ids of the proteins used for generation of the trees have been given in Table 1 (for Arabidopsis), Table 2 (for rice), and Additional file 2: Table S2 (for *S. cerevisiae*, and *H. sapiens*) and Additional file 3: Table S3 (for rest of the species studied).

The CAF1 family showed intriguing phylogenetic relationships (Figures 3A, B and C). Members of the CAF1A subfamily were found to be separated into two major groups with one (marked as ‘III’ in Figure 3A) comprising members from land plants (both basal and higher) and the other (marked as ‘II’ in Figure 3A) with proteins from *S. cerevisiae,* and human (Figure 3A). Putative CAF1A from *C. reinhardtii* formed a separate leaf in the tree (represented as ‘I’ in Figure 3A). Similarly, CAF1B subfamily of proteins comprised three groups (Figure 3B). One group (marked as ‘I’ in Figure 3B) comprised putative CAF1B proteins from *C. reinhardtii* and *S. moellendorffii* while another group (marked as ‘II’ in Figure 3B) possessed CAF1B proteins from yeast and human. The other group (marked as ‘III’ in Figure 3B) possessed members from Arabidopsis, rice, *P. abies*, and *P. patens* (Figure 3B). The other subfamily of CAF1, CAF1C, was found to be most diverse with a clear separation of the plant members into three major groups (Figure 3C)*.* At least one CAF1C protein (excluding splice variants) each from *P. patens*, Arabidopsis, and rice were found in all the three groups. Furthermore, it was interesting to note that while AtCAF1CL1, AtCAF1CL2, AtCAF1CL3, OsCAF1CL1, and OsCAF1CL2 along with one protein each from *C. reinhardtii, P. patens* and *P. abies* and three proteins from *S. moellendorffii* were present together with HsCAF1C in one group (Group III); AtCAF1CL4, AtCAF1CL5, OsCAF1CL3 (both the splice variants) and all three splice variants of Phpat.015G071000 were clustered together with ScCAF1p50 (CAF1C homolog from yeast) in Group II. Group I consisted entirely of putative CAF1C proteins from plants comprising one protein each (and their respective splice variants) from *C. reinhardtii, P. patens, P. abies* and Arabidopsis (AtCAF1CL6), and two proteins each from rice (OsCAF1CL4 and OsCAF1CL5) and *S. moellendorffii* (Figure 3C)*.*

**Figure 4:**
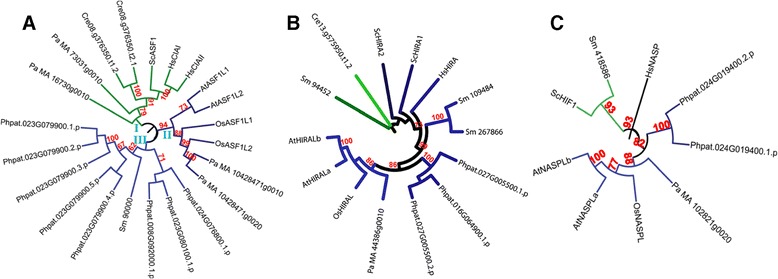
**Phylogenetic analysis of ASF1, HIRA, and NASP families of histone chaperones from diverse eukaryotic taxa.** Phylogenetic trees constructed to show evolutionary distances among the members of ASF1 **(A)**, HIRA **(B)**, and NASP **(C)** families from *Arabidopsis thaliana* (named by prefixing ‘At’), *Oryza sativa* (named by prefixing ‘Os’), *Chlamydomonas reinhardtii* (represented by the prefix ‘Cre’ with the locus id), *Physcomitrella patens* (represented by the prefix ‘Phpat’ with the locus id), *Selaginella moellendorffii* (named by prefixing ‘Sm’), *Picea abies* (named by prefixing ‘Pa’), *Saccharomyces cerevisiae* (named by prefixing ‘Sc’), and *Homo sapiens* (named by prefixing ‘Hs’). Multiple sequence alignment was carried out using ClustalX2 and the tree was constructed using MEGA 6.06 using Maximum Likelihood method and viewed using iTOL. Reliability of the branches was inferred from bootstrap analyses of 1000 replicates. Bootstrap values above 50% have been shown as numbers. Roman numerals show arbitrary numbers given to the phylogenetic groups. Sequence ids of the proteins used for generation of the trees have been given in Table 1 (for Arabidopsis), Table 2 (for rice), and Additional file 2: Table S2 (for *S. cerevisiae*, and *H. sapiens*) and Additional file 3: Table S3 (for rest of the studied species).

The proteins belonging to ASF1 family were found to be divided into three major groups (Figure 4A). ASF1 members from human and yeast along with two proteins from *P. abies* and one protein (two splice variants) from *C. reinhardtii* formed one group (marked as ‘I’ in Figure 4A). Putative ASF1 proteins from Arabidopsis and rice along with one (two splice variants) from *P. abies* comprised another group (marked as ‘II’ in Figure 4A). The third group (marked as ‘III’ in Figure 4A) consisted of putative ASF1 members from *P. patens*, and *S. moellendorffii* (Figure 4A). Phylogenetic tree for the HIRA family suggested relatively lesser divergence in this family. Most of the members formed part of a single major group. Members from the spermatophytic plants (Arabidopsis, rice and *P. abies*) clustered together (Figure 4B). Further, plant HIRA proteins (except that from C. *reinhardtii*, and one from S. *moellendorffii*) were found to be more closely related with each other than with HIRA proteins from yeast and human. In case of NASP family as well, putative members from the spermatophytic plants were found as closely related members of a group (Figure 4C). Besides, most of the plant NASP proteins were found to be closer to HsNASP than to ScHIF1 (NASP homolog in yeast).

**Figure 5:**
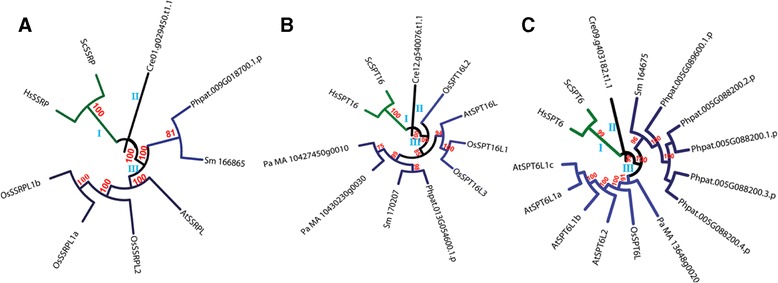
**Phylogenetic analysis of FACT and SPT6 families of histone chaperones from diverse eukaryotic taxa.** Phylogenetic trees were constructed to determine evolutionary distances among the members of each of the two sub-families of FACT family viz. SSRP **(A)**, and SPT16 **(B)**; and SPT6 family **(C)** from *Arabidopsis thaliana* (named by prefixing ‘At’), *Oryza sativa* (named by prefixing ‘Os’), *Chlamydomonas reinhardtii* (represented by the prefix ‘Cre’ with the locus id), *Physcomitrella patens* (represented by the prefix ‘Phpat’ with the locus id), *Selaginella moellendorffii* (named by prefixing ‘Sm’), *Picea abies* (named by prefixing ‘Pa’), *Saccharomyces cerevisiae* (named by prefixing ‘Sc’), and *Homo sapiens* (named by prefixing ‘Hs’). Protein sequences were aligned using ClustalX2 and the tree was constructed using MEGA 6.06 using Maximum Likelihood method and viewed using iTOL. Reliability of the branches was estimated using bootstrap analyses of 1000 replicates. Bootstrap values above 50% have been shown as numbers. Roman numerals show arbitrary numbers given to the phylogenetic groups with lower case letters showing sub-groups. Sequence ids of the proteins used for generation of the trees have been given in Table 1 (for Arabidopsis), Table 2 (for rice), and Additional file 2: Table S2 (for *S. cerevisiae*, and *H. sapiens*) and Additional file 3: Table S3 (for rest of the studied species).

Based on the sub-families, two phylogenetic trees (one each for SSRP and SPT16) were constructed for the FACT family (Figure 5A and B). SSRP proteins were found to be separated in three distinct groups with each group suggesting homology between/amongst members from evolutionarily closer species (Figure 5A). SSRP proteins from human and yeast formed a group (marked as ‘I’ in Figure 5A) distantly related to the other two groups. While the second group (marked as ‘II’ in Figure 5A) possessed a single protein from *C. reinhardtii,* the other group (marked as ‘III’ in Figure 5A) comprised members from rest of the plant species separated into two clusters – one comprising proteins from Arabidopsis and rice, and the other with proteins from *P. patens,* and *S. moellendorffii* (Figure 5A). The phylogenetic tree for the other subfamily of FACT – SPT16, was strikingly similar to that for the SSRP subfamily with proteins from human and yeast constituting a distinct group and separation of members from lower land plants and higher plants (Arabidopsis and rice) into different clusters (Figure 5B). The only major difference was OsSPT16L2 occupying a position distant to other plant SPT16 proteins. Interestingly, phylogenetic tree for another histone chaperone family SPT6 also showed a grouping pattern highly similar to that for the SSRP subfamily (Figure 5C). SPT6 proteins from human and yeast formed a separate group, whereas putative SPT6 proteins from plants were separated into two clusters one comprising proteins from spermatophytic plants and the other with basal land plants.

### Domain architecture and predicted subcellular localization of histone chaperones of Arabidopsis and rice

**Figure 6:**
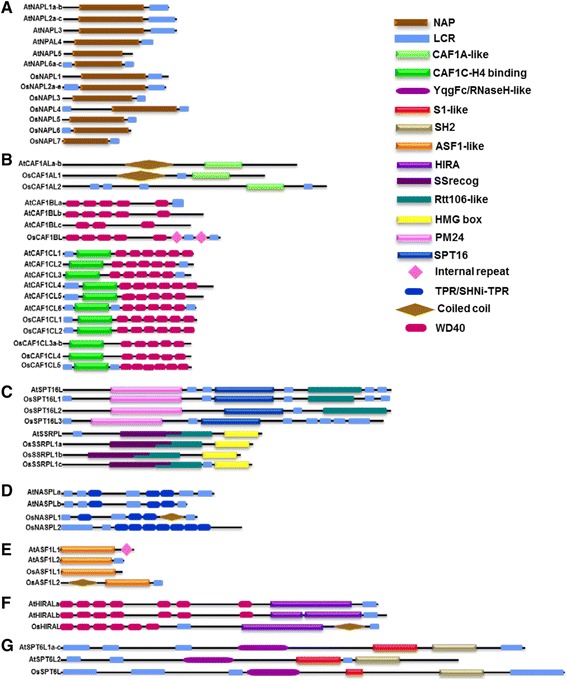
**Domain architecture of histone chaperones from Arabidopsis and rice.** Diagram shows scaled representation of the primary structure of histone chaperones belonging to **(A)** NAP, **(B)** CAF1 (CAF1A, CAF1B, and CAF1C), **(C)** FACT (SPT16 and SSRP), **(D)** NASP, **(E)** ASF1, **(F)** HIRA, **(G)** SPT6 families from Arabidopsis and rice. Positions of various domains along the respective protein sequences have been shown by different shapes as depicted in the key at the right. It is to be noted that each family, except CAF1B, possesses one or more characteristic domains while some families possess domains found in other families of histone chaperones as well. Refer text for the Pfam ids of the domains.

To attribute functions apart from nucleosome assembly/disassembly to different members of various families of histone chaperones, we analyzed their primary structure in detail (see Methods). We found that a common feature of most of the histone chaperones across the diverse seven families is the presence of one or more low complexity regions (LCRs), which are stretches of polypeptide sequence highly rich in one or a few amino acids. Apart from these regions, each of the families (except CAF1B) was found to harbor specific domains while some families possess domains found in other families of histone chaperones as well (Figures 6A-G).

Proteins belonging to the NAP family of both rice and Arabidopsis were found to possess a NAP domain and all but AtNAPL5 have at least one LCR (Figure 6A). The CAF1 family has three sub-families based on the subunits, viz. CAF1A, CAF1B, and CAF1C. The AtCAF1A-like proteins comprise one CAF1A-like domain (Pfam id: PF12253.1) and coiled coil regions (Figure 6B). While OsCAF1AL1 possesses a domain architecture similar to that of its Arabidopsis orthologs, OsCAF1AL2 does not have a coiled coil region and instead possesses four LCR regions apart from one CAF1A-like domain (Figure 6B). All, excluding one (AtCAF1Bc), CAF1B members in both Arabidopsis and rice were found to possess five WD40 domains (PF00400) (Figure 6B). Interestingly, the rice member of this family also possesses two internal repeats. The CAF1C proteins of both Arabidopsis and rice possess a unique CAF1C-H4 binding domain (PF12265.1), apart from five or more WD40 domains (Figure 6B).

Amongst the proteins annotated as SPT16 (a subunit of the FACT complex), each of them possesses a unique domain SPT16/CDC68 (PF08644.4), and Peptidase M24 domain (PM24; PF00557.17). Further, except OsSPT16L3, all harbor an Rtt106-like domain (PF08512.5) (Figure 6C). Interestingly, the PM24 domain has been designated as a metallopeptidase domain; however, when it is found in proteins other than proteases, it has been shown to perform other functions. For instance, PM24 in SPT16 from *S. pombe* functions as a binding module to histones H3 and H4 [23]. The SSRP subunit of FACT in plants was found to possess HMG box (PF00505.12), and Rtt106-like domain (PF08512.5) apart from the characteristic structure-specific recognition domain (SSrecog; PF03531.7) (Figure 6C). The Rtt106-like domain was originally found in Rtt106 histone chaperone-like factor in *S. cerevisiae* which has been found to interact with CAF1C and implicated in heterochromatin-mediated silencing [24]. HMG box is primarily a DNA-binding domain found in several DNA-binding proteins [25]. Though not present in ScPOB3 (SSRP homolog in yeast), it is present in human SSRP (HsSSRP) [4] and we found it in SSRP proteins from Arabidopsis and rice, as well.

We found that the NASP family of proteins in both Arabidopsis and rice characteristically possesses various combinations of loosely defined regions such as LCR, TPR (SM000028), SHNi-TPR (PF10516) – an interrupted form of TPR repeat uniquely found in NASP and related proteins [26], besides coiled coil regions in OsNASPL1 (Figure 6D). Proteins belonging to ASF1-family of histone chaperones in both the plant species studied possess ASF1-like domain (PF04729) and, in some members, LCR, internal repeat, and coiled coil region (Figure 6E). The HIRA family proteins can be classified as WD-repeat proteins as they were found to possess up to seven WD40 domains, and a characteristic HIRA-like domain (PF07569.4) apart from LCRs (Figure 6F). All the members of SPT6 family in rice and Arabidopsis possess YqgFc/RNase-H like (PF14639), S1-like (PF00575), and Src-homology 2 (SH2, PF14633) domains (Figure 6G).

While some histone chaperones are exclusively nuclear, others show nucleo-cytoplasmic shuttling in response to various stimuli [27,28]. Therefore, we analyzed the sequence-based nuclear or cytoplasmic localization of rice and Arabidopsis histone chaperones (see Methods). In Arabidopsis, all the members of SPT16, SSRP, HIRA, ASF1, CAF1A, CAF1B and SPT6 families are predicted to be localized in the nucleus (Table 1). Further, except one member each, all other proteins belonging to the NAP, CAF1C, and NASP families are putatively localized in the nucleus (Table 1). In rice, all the members of SSRP, SPT16, HIRA, NASP, SPT6, CAF1A, and CAF1B families/sub-families and all except three of NAP family, all but four of CAF1C sub-family and one of the two ASF1 family proteins are predicted to be localized in the nucleus (Table 2). To further attribute the intracellular localization to the presence of elements in the primary structure of these proteins, we analyzed the sequences for the presence/absence of nuclear localization signal (NLS) and nuclear export signal (NES) (see Methods). We found that while many histone chaperones predicted to be localized in the nucleus possess putative NLS, others are not predicted to possess NLS (Tables 1 and 2). Besides, many histone chaperones also have putative NES in their sequences (Tables 1 and 2), suggesting that they may show nucleo-cytoplasmic shuttling.

### Expression profiling during plant development and across different plant tissues shows differential transcriptional regulation of a few histone chaperones

**Figure 7:**
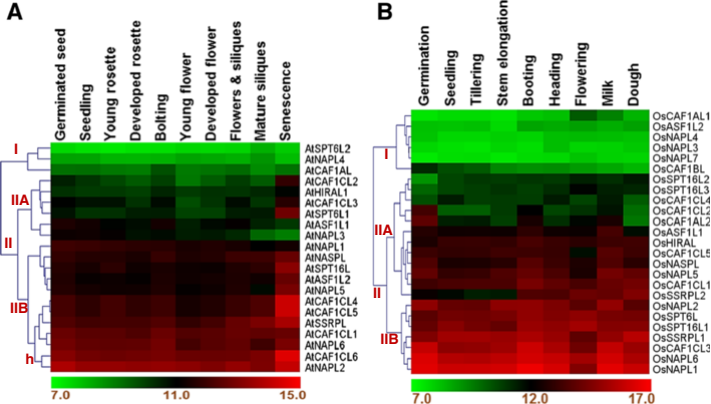
**Expression pattern of histone chaperones in Arabidopsis and rice during development.** Heat maps show microarray-based expression profile of histone chaperones from Arabidopsis **(A)** and rice **(B)** at various developmental stages indicated at the top of each of the map. ‘Milk’ and ‘Dough’ represent stages of seed development in rice. Hierarchical clustering using weighted average linkage method and Euclidean distance metric was used to generate the heat maps. Color bars at the bottom of each of the heat maps show the corresponding scale for log_2_ expression, with green representing lowest expression and red representing the highest. Roman numerals followed by uppercase letters represent position of some major clusters as referred to in the text. ‘h’ represents a specific sub-cluster as part of the cluster IIB.

To gain some insights into the possible function of histone chaperones during plant development, we analyzed the microarray-based expression pattern of histone chaperones at different stages of development in the two plant species studied (see Methods). We found three distinct gene expression patterns – consistent low expression, constant high expression and developmental stage-specific regulation of expression (Figure 7A and B). In Arabidopsis, *AtSPT6L2*, *AtNAPL4*, and *AtCAF1AL* showed a low level of expression across all the developmental stages (marked as cluster I in the heat map in Figure 7A). On the other hand, *AtNAPL2* and *AtCAF1CL6* showed a high expression which remained fairly constant during development (marked as ‘h’ in Figure 7A). Other genes were found to be differentially expressed across various developmental stages. Amongst those, the most notable ones include all the members of AtCAF1CL sub-family (except *AtCAF1CL1*), *AtSSRPL*, *AtSPT16L* and *AtSPT6L1* which were expressed at their highest level during senescence. Further, the expression of *AtASF1L1* was found to be highest at bolting and in germinated seeds. Besides, the expression of *AtASF1L2* and *AtNAPL5* varied considerably across the developmental stages (Figure 7A).

In rice, we observed a much diverse pattern of expression of histone chaperones during development (Figure 7B). The genes which showed very low expression throughout the developmental stages studied were *OsCAF1AL1*, *OsASF1L2*, *OsNAPL4*, *OsNAPL3*, *OsNAPL7*, and *OsCAF1BL* (marked as cluster I in the heat map in Figure 7B) while those which showed moderately low expression were *OsSPT16L3*, *OsSPT16L2*, and *OsCAF1CL4*. Amongst the genes which showed a fairly high expression throughout development were *OsNAPL1*, *OsNAPL6*, *OsCAF1CL3*, *OsSSRPL1*, *OsSPT16L1*, *OsSPT6L*, and *OsNAPL2* (marked as cluster IIB in the heat map in Figure 7B). *OsCAF1AL2* and *OsCAF1CL2* were expressed at their highest level only during seed germination stage after which the expression was lower. Interestingly, *OsSSRPL2* showed a lower expression during the vegetative stages and much higher during the reproductive stages (booting onwards) (Figure 7B). Since the genes known to play a role in vegetative-to-flowering transition have been found to show a similar characteristic expression pattern [29], it suggests that OsSSRPL2 and hence the FACT complex might play a role in this important transition during the life cycle. This possibility, however, requires validation using overexpression and knockout/knockdown strategies.

**Figure 8:**
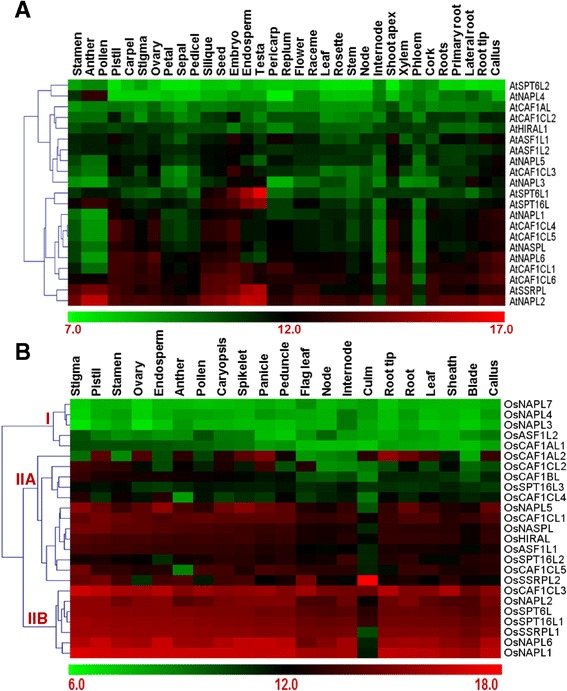
**Microarray-based expression profile of histone chaperones in Arabidopsis and rice across different tissues.** Heat maps show the expression profile of histone chaperones from Arabidopsis **(A)** and rice **(B)** across different tissues of the plants. Log_2_-transformed mean signal intensity values for each of the genes in the respective plant tissues were used to generate the heat maps. Average linkage method and Euclidean distance metric were used for clustering in the heat map. Color bars at the bottom of each of the heat maps show the corresponding scale for log_2_ expression. Roman numerals followed by uppercase letters represent position of some major clusters as referred to in the text.

Next we addressed whether some histone chaperones show tissue-specific expression pattern or they are ubiquitously expressed across all plant tissues. For this, we analyzed their expression across different tissues of Arabidopsis and rice. We found that while several others show a near constant expression pattern either low or high, higher expression of some histone chaperones is restricted to particular plant tissues. For instance, in Arabidopsis, we found that *AtSPT6L1* is specific to some parts of the seed and silique (Figure 8A). Besides, we found that while *AtNAPL2, AtSSRPL*, and *AtSPT16L* are expressed at a higher level in seed and male floral parts, higher expression of *AtCAF1CL6*, *AtCAF1CL1, AtNAPL6*, *AtNASPL*, *AtCAF1CL5*, and *AtCAF1CL4* was found in parts of seed and female floral parts (Figure 8A). In Rice, we found that while several histone chaperones show near constant expression pattern (at either a low or a high level – marked as cluster I and IIB, respectively in the heat map in Figure 8B), a few members show tissue-specific expression (Figure 8B). Uniquely, *OsSSRPL2* shows strongest expression in the culm tissue. Besides, *OsCAF1AL2* showed a higher expression in female floral organs, panicles, spikelets, and root and its parts (Figure 8B).

### Transcriptional regulation of some histone chaperones under biotic and abiotic stress conditions suggests their role in stress response

**Figure 9:**
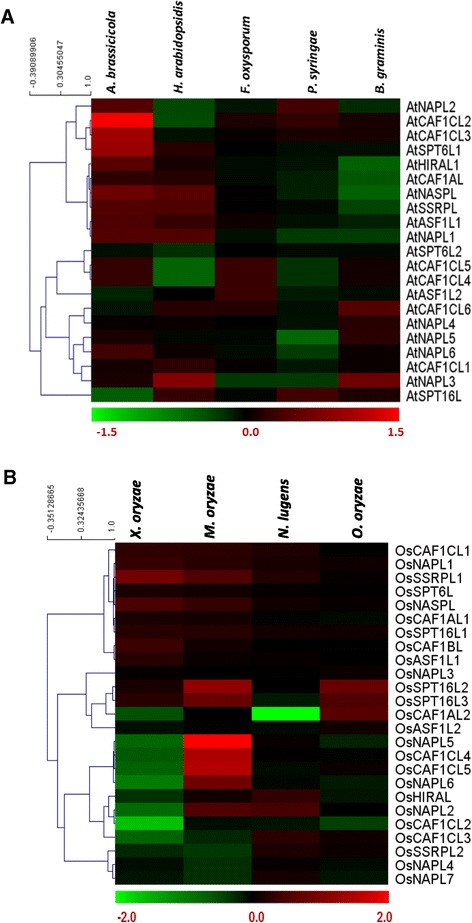
**Expression profile of histone chaperones in Arabidopsis and rice under various biotic stress conditions.** Heat maps show the microarray-based expression pattern of histone chaperones from Arabidopsis **(A)** and rice **(B)** under conditions of infection with different pathogens as indicated at the top of the heat map. Color bars at the bottom of each of the heat maps show the corresponding scale for log_2_ fold change in expression. Heat maps were generated using hierarchical clustering for which weighted average linkage method and Pearson correlation distance metric were used. Genes which showed more than two-fold up- or down-regulation {log_2_(fold change) ≥ 1, or log_2_(fold change) ≤ -1, respectively; Student’s t-test p < 0.05} were considered to be showing significant differential regulation of expression in the respective conditions.

To gain insight into their probable role in both biotic and abiotic stress response, we analyzed the expression of histone chaperones under such stress conditions using publicly available microarray databases. We found significant differential regulation {log_2_(fold change) > 1 for upregulated genes, log_2_(fold change) < -1 for downregulated genes; and two-tailed Student’s t-test: p < 0.05} of several of the histone chaperones under one or the other stress conditions (Figures 9A and B). For expression analysis under biotic stress conditions, we selected *Alternaria brassicicola, Hyaloperonospora arabidopsidis, Fusarium oxysporum, Pseudomonas syringae,* and *Blumeria graminis* as some biotic stress agents for Arabidopsis; and *Xanthomonas oryzae*, *Magnaporthe oryzae*, *Nilaparvata lugens*, and *Oligonychus oryzae* as some biotic stress agents for rice*.* In Arabidopsis, *AtCAF1CL2*, *AtCAF1CL3*, and *AtSPT6L1* were found to be upregulated under conditions of infection by *A. brassicicola* (Figure 9A). In rice, while *OsCAF1CL2* and *OsNAPL6* were found to be downregulated under *Xanthomonas oryzae* infection, *OsSPT16L2*, *OsNAPL5, OsCAF1CL4*, and *OsCAF1CL5* were all found to be upregulated in *Magnaporthe oryzae* (rice blast fungus) infection (Figure 9B). Further, *OsCAF1AL2* was found to be considerably downregulated under conditions of infection of the brown planthopper *Nilaparvata lugens* (Figure 9B)*.*

**Figure 10:**
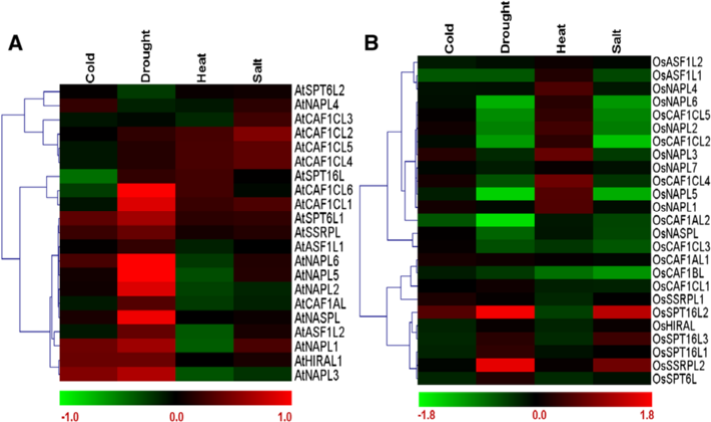
**Microarray-based expression profile of histone chaperones in Arabidopsis and rice under various abiotic stress conditions.** Heat maps show the expression pattern of histone chaperones from Arabidopsis **(A)** and rice **(B)** under various abiotic stress conditions namely cold, drought, heat and salinity as obtained via expression analysis using microarray data. Color bars at the bottom of each of the heat maps show the corresponding scale for log_2_ fold change in expression, with green representing downregulation and red signifying upregulation. Heat maps were generated using hierarchical clustering for which weighted average linkage method and Pearson correlation distance metric were used. Genes which showed more than two-fold up- or down-regulation {log_2_(fold change) ≥ 1, or log_2_(fold change) ≤ -1, respectively; Student’s t-test p < 0.05} were considered to be showing significant differential regulation of expression in the respective conditions.

We further analyzed the expression of histone chaperones in both the species under four abiotic stress conditions namely cold, drought, heat and salt. In Arabidopsis, we found *AtCAF1CL6, AtNAPL6*, *AtNAPL5*, and *AtNASPL* to be upregulated under drought conditions (Figure 10A). In rice, we found a total of eight genes namely *OsCAF1AL2*, *OsNAPL5*, *OsNAPL6, OsCAF1CL5*, *OsCAF1CL2*, *OsCAF1BL*, *OsSPT16L2*, and *OsSSRPL2* showing differential expression under one or the other abiotic stress conditions (Figure 10B).

### qRT-PCR based expression analysis of eight histone chaperones in both sensitive and tolerant rice genotypes confirms their altered expression under abiotic stress conditions

**Figure 11:**
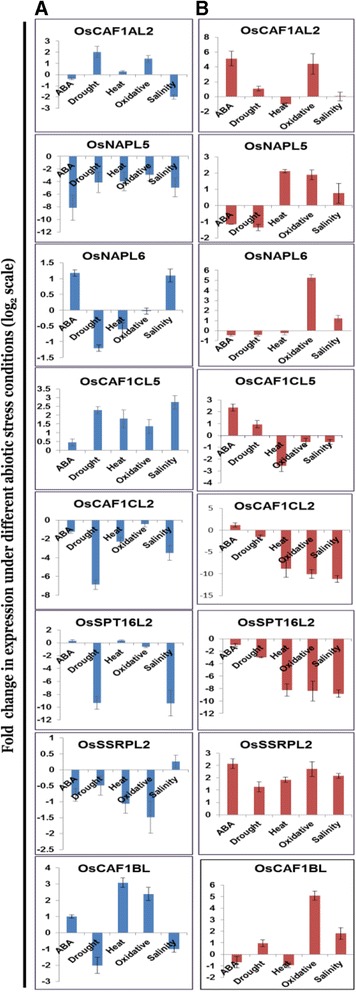
**qRT-PCR confirms the differential expression of eight rice histone chaperone genes under various abiotic stress conditions.** Abiotic stress-responsiveness of the genes which were found to show altered expression in one or more abiotic stresses via microarray-based expression analysis was further studied by qRT-PCR in both stress-sensitive and salinity-tolerant genotypes. Bar graphs depict mean fold change (log_2_ scale) in expression of abiotic stress-regulated histone chaperones in contrasting genotypes – moderately stress sensitive IR64 **(A)**, and salt stress tolerant Pokkali **(B)** under various abiotic stress conditions – ABA, drought, heat, oxidative, and salinity, as obtained via expression analysis using qRT-PCR. Error bars show ± standard deviation, n = 3. Names of the genes have been mentioned at the top of each of the bar graphs. Each of the eight genes were found to be differentially regulated under at least one of the stress conditions in both the genotypes, with most of them exhibiting multiple stress-responsive nature.

To further validate the altered expression of some histone chaperones of rice under one or more abiotic stress conditions as observed in microarray-based expression profiling, we carried out qRT-PCR and analyzed the expression of eight histone chaperone genes. Further, to comment upon the comparative expression profile between contrasting genotypes, we chose a moderately stress sensitive (IR64) and a salinity stress tolerant (Pokkali) rice genotype and analyzed the expression under various abiotic stress conditions namely drought, heat, oxidative, salinity as well as ABA (a phytohormone functioning in stress response) treatment. The fold change in expression observed under these conditions has been shown as bar graphs (Figure 11A and B for IR64 and Pokkali genotypes, respectively). Most of the genes encoding histone chaperones showed differential expression in response to multiple abiotic stresses.

In IR64, *CAF1AL2* was found to be upregulated under drought and oxidative stress conditions while it was downregulated under salinity stress condition. In Pokkali also, it was found to be upregulated in oxidative stress conditions as well as under ABA stimulus. *OsNAPL5* was found to be downregulated in all the five stress conditions in IR64, while in Pokkali it was downregulated only in ABA and drought stress treatments. In heat and oxidative stress conditions, *OsNAPL5* was found to be upregulated in Pokkali. The other member of NAP family – *OsNAPL6* was found to be downregulated under drought conditions and upregulated in ABA and salt treatments in the sensitive genotype IR64. In Pokkali as well, *OsNAPL6* was found to be upregulated in salinity, wherein it was also upregulated in oxidative stress conditions. *OsCAF1CL5* was found to be upregulated in drought, heat, oxidative stress and salinity in IR64, while in Pokkali it was upregulated upon ABA treatment and downregulated in heat. *OsCAF1CL2* was downregulated in ABA, drought, heat, and salinity stresses in IR64, whereas in Pokkali it was downregulated in all the stresses except ABA, where it was upregulated. *OsSPT16L2* was found to be considerably downregulated in drought and salinity in both IR64 and Pokkali. Besides, in Pokkali, it was also downregulated in heat and oxidative stresses. While *OsSSRPL2* was upregulated in all the studied stresses in Pokkali, in IR64 it was found to be downregulated under heat stress conditions. *OsCAF1BL* showed a complex pattern of expression in the sensitive genotype IR64. It showed upregulation in heat and oxidative stress but downregulation in drought and salinity. In Pokkali, *OsCAF1BL* was upregulated in both oxidative and salinity stress conditions. The differential regulation of gene expression under multiple abiotic stress conditions in contrasting genotypes points towards the role of these histone chaperones in abiotic stress response in plants, which remains to be functionally validated in future.

## Discussion

Regulation of gene expression is a fundamental process in a cell and plays a critical role in physiological and developmental processes in plants. Further, plants being sessile regulate the expression of hundreds and thousands of genes in order to successfully respond to stimuli generated by biotic and abiotic stresses. Gene expression and its regulation involve a network of numerous cellular processes and factors involved in them. By altering the DNA accessibility via eviction and deposition of histones onto the DNA, histone chaperones represent an important regulatory hub in the gene expression webs and hence can potentially exert considerable influence on developmental and physiological processes in plants. In contrast to their yeast and human counterparts, however, plant histone chaperones remain poorly studied and their physiological role in plants remains elusive. Therefore, there is a need to comprehensively identify and dissect the roles of histone chaperones in plants.

We identified and classified histone chaperones from two model plants – rice and Arabidopsis, and studied their phylogenetic relationship with histone chaperones from other organisms including yeast, human, algae, basal land plants and one conifer. Our finding that plant histone chaperones comprise majorly multi-membered families partly due to some events of segmental duplication leading to gene-family expansion suggests towards interesting links between histone chaperones and evolution and divergence of dicots and monocots. Our analysis indicates that while most duplication events might be common to dicots and monocots, we do find dicot-specific and monocot-specific expansion of some histone chaperone families (Figure 1). Segmental duplication has been considered to be a common process in plants leading to expansion of gene families [30,31], and histone chaperones present no exception.

Phylogenetic analyses of histone chaperones from diverse organisms such as yeast, algae, bryophyte, pteridophyte, gymnosperm, angiosperms, and human indicate that most histone chaperones from yeast and human, except those belonging to NASP and HIRA families and the CAF1C sub-family, are more closely related to each other than to histone chaperones from plants (Figures 2, 3, 4, 5). Besides, histone chaperones (excluding those belonging to NAP, ASF1, and CAF1C) from the alga *Chlamydomonas reinhardtii* formed a distinct group within their respective families indicating their distant evolutionary relationship with counterparts from other species (Figures 2, 3, 4, and 5). The phylogenetic trees further indicate interesting possibilities about the link between evolution and function of histone chaperones. For instance, the highly similar pattern of evolution of the two subfamilies (SSRP and SPT16) of the FACT family (Figure 5A and B) suggests that since the two subunits (SSRP and SPT16) of the FACT multi-subunit complex often function together, their evolution, possibly, might have been on similar lines. Another intriguing example is the CAF1C subfamily, plant members of which showed diversity both across species (interspecific) and within a single species (intraspecific). Plant CAF1C proteins from the same species are separated in distinct phylogenetic groups (Figure 3C). Most of the CAF1C proteins from the studied plant species were found to be separated in two phylogenetic classes. However, CAF1C members from rice, Arabidopsis and *P. patens* formed part of three different phylogenetic classes. Thus, CAF1C sub-family in plants shows both intra- and inter-specific variation. This suggests a possible functional divergence in the CAF1C subfamily in plants; that is not found in yeast and human. Because this sort of divergence is absent in CAF1A and CAF1B (Figure 3A and B), divergence in the CAF1C subfamily in plants might contribute to regulate the function of CAF1 complex via replacing one CAF1C subunit with another in the CAF1 multi-subunit complex. Since the CAF1 multichaperone complex is involved in histone deposition during replication and repair of DNA [5], this possible mechanism may serve as a means to respond to various stimuli during these processes. The replacement of components of a multi-subunit complex in order to regulate biological function has been found to be a feature of some macromolecular complexes, the most striking example being histones as part of the histone octamer [32]. However, validating the possibility, as to if the activity of the CAF1 complex is regulated by replacing one CAF1C subunit with another, warrants further biochemical evidence and remains to be worked out in future.

Histone chaperones are also classified based on their histone binding specificity as most of them show preference towards a particular class of histones, either H2A/H2B or H3/H4 [5]. Amongst the NAP family proteins, HsSET shows preferential binding towards H3-H4 class of histones [33], while ScNAP1 and other NAP-proteins from human are considered to be H2A-H2B chaperones [5,34]. The clustering of AtNAPL5, AtNAPL6, OsNAPL5, and OsNAPL6 (and at least one putative NAP protein from each of the studied plant species) with HsSET, while other members constituting a different clade with NAP members from yeast (ScNAP1) and human (Figure 2) indicated functional divergence in the NAP family in higher eukaryotes. This suggests similar histone-specificity for the corresponding homologs in Arabidopsis and rice, with Group I and II possibly being H3/H4- and H2A/H2B-specific, respectively (Figure 2). Further, our results explain the differential phenotypic information obtained via mutant analysis in previous studies. In Arabidopsis, it has been shown that double mutants of *AtNAPL6* (*NRP1*) and *AtNAPL5* (*NRP2*) which are clustered with HsSET in the tree (Figure 2), show growth defect in roots [35] while the triple mutant of *AtNAPL1*(*AtNAP1;1*), *AtNAPL2* (*AtNAP1;2*) and *AtNAPL3* (*AtNAP1;3*) shows sensitivity to ultraviolet radiation [18]. This indicates that apart from being evolutionary distinct, these two groups of the NAP family perform different physiological functions in plants.

Primary structure analysis of histone chaperones in plants reveals that a common feature of most of the histone chaperones is the presence of one or more low complexity regions – LCRs (Figure 6). LCRs are characterized by low sequence diversity and possess the ability to expand in a shorter time via slippage during replication [36], thus generating diversity in the protein families based on the number of LCRs. We observed that in case of many histone chaperones, the LCRs present at the C-terminus are rich in acidic residues aspartate (D) and glutamate (E) (Figure 6; Additional file 4: Figure S1, Additional file 5: Figure S2, Additional file 6: Figure S3, Additional file 7: Figure S4, Additional file 8: Figure S5, Additional file 9: Figure S6, Additional file 10: Figure S7, Additional file 11: Figure S8, Additional file 12: Figure S9, Additional file 13: Figure S10), conserved D/E residues are shown in purple in the alignments). Portions of these LCRs are known to be sites of post-translational modifications [37], and hence may be involved in modulating interaction and the specificity of interaction with other proteins including histones [38]. Furthermore, all the families of histone chaperones except CAF1B possess at least one domain not found in other families. CAF1B is a unique histone chaperone insofar as it possesses only WD40 domain in multiple copies across its sequence (Figure 6B). WD40 domain is also present in other proteins including histone chaperones CAF1C and HIRA (Figure 6B and F), and has been considered to be majorly a eukaryotic domain functioning in protein-protein interaction [39]. That CAF1B, CAF1C, and HIRA are all known to function as part of macromolecular complexes [4], elucidates the importance of the presence of these repeats in them, since protein-protein interactions are the very basis of the assembly of such complexes.

Several mechanisms such as regulation at the transcriptional, post-transcriptional, translational or post-translational level and interaction with other macromolecules influence the final activity and function of a protein. Amongst these, transcriptional regulation is a major means to regulate the cellular levels and hence the activity of an encoded protein. Consistently, gene expression-based studies in plants have shown that transcript profiles of genes usually correlate well with their role in physiology and development [29,40]. Hence, studying the transcript profiles of genes which do not have well described role during the course of a plant’s life cycle, may provide meaningful insights into their function. We, therefore, analyzed the expression of histone chaperones of both Arabidopsis and rice during development and across different plant tissues. Because histone chaperones serve several vital functions inside the cell, their levels are not expected to vary considerably at different stages in life cycle and across various tissues. However, in contrast to yeast, plants possess multiple histone chaperones in most of the families and mutant analyses have shown that cellular function of the members of some of the families is redundant [19,35,41]. This gives a scope for modulating the expression of a few histone chaperones in order to respond to developmental and stress signals via altering chromatin accessibility at the target loci. In our analysis, we found that while several histone chaperones maintain their transcript levels, either high or low, throughout development and across different tissues, many others are expressed at a higher level at a particular developmental stage or in specific plant tissues (Figures 7 and 8).

The genetic reprogramming associated during developmental processes and formation of different tissues from the embryo requires coordinated expression of specific suites of genes. Epigenetic regulation of gene expression is an important means of controlling cellular levels of gene products and maintaining both intra-generational and trans-generational memory [42]. Since histone chaperones are important players in these processes, our data suggest that histone chaperones may be involved in the epigenetic programming and reprogramming associated with development and formation of organ identity. Though not always the case, many factors regulating gene expression and functioning to contribute towards grain yield have been shown to be expressed differentially at various developmental stages [29]. Nevertheless, differential expression of a gene across different panicle development stages *per se* is only an indication of its probable function in contributing towards grain yield. Hence, even though we have found that a few histone chaperones are differentially expressed during reproductive phases, further detailed studies using tools of functional genomics are required to delineate the contribution, if any, of such histone chaperones towards grain yield.

Stress response requires altered expression of a large number of genes [43-45]. Switching on and off the expression of so many genes under stress conditions is associated with the action of several transcription factors like DREB, LEA, WRKY, AP, DST and NAC [46-48]. However, the action of transcriptional activators requires a transcriptionally competent chromatin state and that of repressors is associated with a restrictive chromatin conformation. Histone chaperones, due to their ability to assemble/disassemble nucleosomes, function together with other epigenetic factors like ATP-dependent chromatin remodeling factors, HDACs, and HATs to alter the transcriptional competence of a chromatin region [49]. Hence, histone chaperones can potentially play a major role in stress response in plants. There is a paucity of reports validating the possible function of histone chaperones in stress response in plants. Nevertheless, the expression profile obtained in our study shows that some histone chaperones are differentially regulated under one or more biotic and abiotic stress conditions while the levels of others remain unchanged. In agreement with the results of the present study (Figure 10A), recently, it has been shown via mutant analyses that Arabidopsis histone chaperones belonging to ASF1 family play a role in transcriptional activation in response to heat stress [50]. Besides, previously, it has been found using expression values from public databases that *ASF1B* in Arabidopsis (*AtASF1L1*) is downregulated under heat stress conditions [19]. Our observations (Figures 9, 10, 11) supported by these findings suggest that altered levels of a few histone chaperones, including ASF1, may be instrumental in promoting or restricting DNA accessibility at stress-responsive regions of DNA, depending on whether the chaperone in question primarily functions in eviction or deposition of histones. Considering that gene expression is a complex interplay of hundreds of diverse factors, to facilitate a holistic understanding of gene regulation during stress response, it is imperative to generate a comprehensive picture taking into account both genetic as well as epigenetic factors, including histone chaperones.

Several stresses are associated with DNA damage and in order to survive under stressful conditions, the damaged DNA must be repaired [51]. DNA repair machinery requires the aid of histone chaperones in order to gain access to the damaged DNA [52,53]. Therefore, for an efficient DNA damage response, altered expression and function of histone chaperones might be required. Previously, it has been shown that double mutation in *AtNAPL6* (*AtNRP1*) and *AtNAPL5* (*AtNRP2*) is associated with a down regulation of DNA repair components [35]. In our study, genes for both these histone chaperones were found to be upregulated during drought stress conditions (Figure 10). These findings together indicate that AtNAPL5 and AtNAPL6 may positively regulate the expression of some components of the nucleotide excision repair machinery and thus possibly play an indirect role in DNA repair. However, precisely which histone chaperones are involved directly in DNA repair pathways in plants is not known, to date, and further interaction studies are required to fish out the histone chaperones interacting with the DNA repair machinery.

## Conclusions

**Figure 12:**
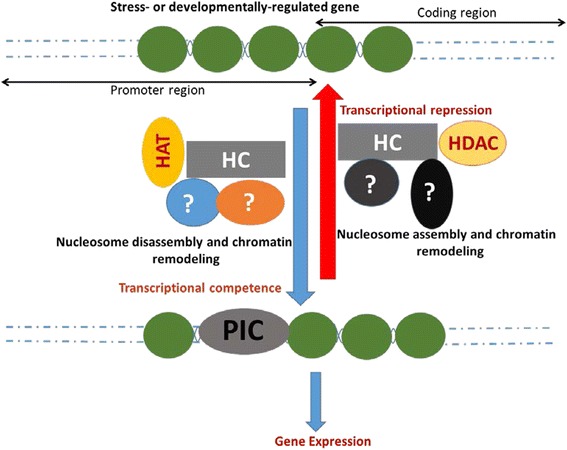
**Hypothetical model for histone chaperone function during development and stress response in plants.** Schematic diagram shows a simplified model for function of histone chaperones at developmentally-regulated and stress-regulated genetic loci in plants. Histone chaperones (marked as HC) with the coordinated action of other factors (shown as shapes with question marks) including those involved in chromatin remodelling, DNA demethylation, histone methylation, and histone acetylation by HATs (histone acetyl transferases) may promote transcriptional competence in the direction of the downward blue arrow. In this direction, histone chaperones primarily aid the transient eviction of histones from the chromatin template leading to the assembly of pre-initiation complex (PIC) followed by transcription. On the other hand, in the reverse direction (shown by upward red arrow) histone chaperone may function to deposit histones onto the DNA template. The resulting enhanced nucleosome occupancy, which may also require the functioning of histone deacetylases (HDACs), some histone methyl transferases and the DNA methylation machinery apart from histone chaperones, leads to transcriptional repression. Precisely which factors cooperate with histone chaperones resulting in either of the two contrasting outcomes (transcriptional competence or repression), during stress response and developmental programming and reprogramming in plants, remains to be worked out and hence are shown as shapes with question marks. Broken lines represent the classic nucleosome structure in continuation.

By affecting the accessibility of DNA for various DNA-related processes, histone chaperones represent an important class of ‘master regulators’ which can modulate the expression of several genes. Therefore, histone chaperones can potentially play a key role during physiological and developmental processes in plants. However, histone chaperones have not been well studied in plants and their precise number, architecture, and transcriptional regulation remain poorly understood. Our study, for the first time has identified the members of all the seven families of histone chaperones in two model plants – Arabidopsis and Rice. Our attempt to trace the evolutionary trajectory of histone chaperones in plants by including representative species from every major plant group for a phylogenetic reconstruction has provided insights with intriguing biochemical and functional implications which remain to be studied in a greater detail. Further, the expression pattern during both development and stress response, obtained in the present study, suggests novel roles for histone chaperones vis-à-vis these processes. Based on the cellular role of histone chaperones and the results of the present study, we hypothesize that histone chaperones, in conjunction with other factors involved in regulation of gene expression, play an important and possibly a regulatory role in stress response and during development in plants (Figure 12). Future studies may aim to functionally characterize the differentially regulated histone chaperones furthering our understanding of the underlying regulatory networks of gene expression and delineating the precise role of histone chaperones therein.

## Methods

### Identification and nomenclature of histone chaperones in Arabidopsis and rice

To identify the histone chaperones in Arabidopsis and rice, Hidden Markov Model (HMM) profiles unique to NPM (PF03066), NAP (Pfam accession no. PF00956), CAF1A (PF12253), CAF1C (PF12265), SPT6 (PF14632 and PF14639), ASF1 (PF04729), HIRA (PF07569), NASP (PF10516), SPT16 (PF08644) and SSRP (PF03531) families were retrieved from Pfam database version 26.0 [54] (http://pfam.sanger.ac.uk/) and thereafter searched against the respective model plant protein databases TAIR10 (http://www.arabidopsis.org/) for Arabidopsis, and TIGR Rice 7.0 [55] (http://rice.plantbiology.msu.edu/) for rice, using the HMMER 3.0 software [56] (http://hmmer.janelia.org/). The proteins thus identified, after using the inclusion threshold, were verified using both Pfam and SMART [57] (http://smart.embl-heidelberg.de/) and the members, if any, not possessing the respective unique domains were discarded. The purpose of this post-genome wide identification step is to identify false positives, like transposable elements, containing partial domain sequences [58,59].

The absence of NPM-related proteins in thirty-seven diverse plant species with sequenced genomes was confirmed using PLAZA 3.0 Dicot and PLAZA 3.0 monocot comparative genomics platforms (http://bioinformatics.psb.ugent.be/plaza/) [60] using BLASTp at a low stringency threshold (expect: 1). In case of CAF1B sub-family, which was not found to possess any unique domain and instead possesses multiple copies of WD40 domains (which are also present in many other protein families), we utilized a BLASTp approach to search for CAF1B members in the two plants. A stringent BLASTp search (expect: 1e-30) was carried out using HsCAF1B, and ScCAF1B, individually against the respective plant genome databases (TAIR 10 for Arabidopsis, and TIGR Rice v7.0 for rice) via stand-alone BLASTP 2.2.27+. The reason for using a rather conservative BLASTp-based search was to avoid false positives and incorrect gene annotations. While searching genomes for the presence of members of a protein family, false positives have been considered to be more undesired than false negatives in most cases [61].

For nomenclature, we have used the standard practice based on the HMM score [30]. For example, in the NAP family, OsNAPL2a would mean that it is the second member of the rice NAP family and closest among the splice variants coded by *OsNAPL2* gene. We have purposefully suffixed ‘L’ (for ‘like’) with the name of the family due to the relatively low complete-sequence similarity to the corresponding member from human/yeast which has been taken as the characteristic member of the respective family.

### Sub-cellular localization, primary sequence analysis and domain architecture of histone chaperones from Arabidopsis and rice

The primary structure of histone chaperones in both the plant species was analyzed to predict their intracellular localization using CELLO [62] (http://cello.life.nctu.edu.tw/). Further, the presence of nuclear localization signal (NLS) and nuclear export signal (NES) was predicted using cNLS mapper [63] (http://nls-mapper.iab.keio.ac.jp) and NetNES v1.1 [64] (http://www.cbs.dtu.dk/services/NetNES-1.1/), respectively, using default parameters. Data for the predicted isoelectric point (pI) and polypeptide molecular weight (Mw) as given in Tables 1 and 2 were retrieved using the respective genome browsers (TAIR10 and TIGR Rice v7.0 for Arabidopsis and rice, respectively).

The sequences were further used for predicting the domain architecture using Simple Modular Architecture Research Tool, SMART [57] and Pfam version 26.0 databases. The representative domain structures of various histone chaperones was drawn using gplots package of open source R statistical software using R scripts and the same were redrawn using MS Powerpoint to improve clarity.

### Chromosomal distribution of genes encoding histone chaperones in Arabidopsis and rice and detection of duplication events

The identified histone chaperones were mapped on respective *Arabidopsis* and rice chromosomes using chromosome coordinates from the respective genome databases TAIR10 and TIGR Rice v7. To determine whether some genes (encoding histone chaperones) are present on duplicated segments of chromosomes in Arabidopsis and rice, we used the Plant Genome Duplication Database (http://chibba.agtec.uga.edu/duplication/index/home [65], which sources its data from the respective genome browsers – TAIR for Arabidopsis, and RAP (http://rapdb.dna.affrc.go.jp/) for rice. The data for collinear blocks was retrieved using the URL http://chibba.agtec.uga.edu/duplication/index/downloads by selecting *O. sativa* vs. *O. sativa* (for rice), and (*A. thaliana* vs. *A. thaliana*) for Arabidopsis. The loci for various histone chaperones were then searched manually in the data for collinear blocks. For rice loci, RAP-DB id converter (http://rapdb.dna.affrc.go.jp/tools/converter) was used to convert MSU ids to RAP-DB ids and vice versa. The scaled position of genes on respective chromosomes and segmental duplication events has been drawn using gplots package of open source R software and the pictures were modified in MS PowerPoint to enhance readability.

### Multiple sequence alignment and phylogenetic analysis of histone chaperones from diverse eukaryotes

In order to determine the phylogenetic relation between histone chaperones from diverse eukaryotic organisms, we first identified putative histone chaperones from *Chlamydomonas reinhardtii* (a green alga), two basal land plants viz. *Physcomitrella patens* (a bryophyte)*, Selaginella moellendorffii* (a pteridophyte), and a conifer – *Picea abies*. The methodology followed was similar to that followed for identification of histone chaperones from rice and Arabidopsis. Phytozome v10 [66] (http://phytozome.jgi.doe.gov/pz/portal.html) was used to retrieve the sequence data for *C. reinhardtii*, and the two basal land plants. The resources corresponding to the genome sequences are: http://phytozome.jgi.doe.gov/pz/portal.html#!info?alias=Org_Creinhardtii [67], *Physcomitrella patens* v3.0, DOE-JGI, http://phytozome.jgi.doe.gov/pz/portal.html#!info?alias=Org_Ppatens [68,69], and http://phytozome.jgi.doe.gov/pz/portal.html#!info?alias=Org_Smoellendorffii [70]. For *P. abies*, sequence data was retrieved using ConGenIE [71] (http://congenie.org/). Uniprot (http://www.uniprot.org/uniprot/) was used to retrieve sequences of various histone chaperones from yeast and human.

Multiple sequence alignments for all protein sequences from *Arabidopsis thaliana* (Table 1), *Oryza sativa* (Table 2)*, Homo sapiens* (Additional file 2: Table S2)*, Saccharomyces cerevisiae* (Additional file 2: Table S2) and *C. reinhardtii*, *P. patens*, *S. moellendorffii*, and *P. abies* (Additional file 3: Table S3) were performed using ClustalX2 using default parameters and the alignments were viewed using Jalview [72]. The sequence alignments were then used for phylogenetic reconstruction for each of the histone chaperone families/subfamilies by the Maximum Likelihood method using MEGA6.06 software [73]. While generating phylogenetic trees in MEGA6.0.6, the widely used empirically-derived Jones-Taylor-Thornton substitution model [74] was selected for calculating probabilities of change along branches. The reliability of branches was inferred from a bootstrap analysis of 1000 replicates. Rest of the parameters selected in MEGA 6.0.6 were default. The final phylogenetic tree was then drawn and viewed using iTOL [75] (http://itol.embl.de/). The images were, then, manually edited to improve readability using MS PowerPoint.

### Microarray-based expression analysis

For microarray-based expression profiling, publicly available expression data for the Rice and Arabidopsis Affymetrix microarray platform (51 K and 22 K Affymetrix gene chips, respectively) were used. For studying expression pattern at various developmental stages and in different plant tissues, the normalized and curated log_2_-transformed signal intensity values on the respective arrays were retrieved using Genevestigator database tool [76,77] (https://www.genevestigator.com/gv/plant.jsp) using default parameters as done previously [29]. We could find specific microarray probe-sets for all the genes for histone chaperones of Arabidopsis and rice except *AtCAF1BL*. Expression datasets used for generating the heat maps have been provided as additional tables (Additional file 14: Table S4, Additional file 15: Table S5, Additional file 16: Table S6, Additional file 17: Table S7).

For analyzing expression levels under different biotic and abiotic stresses, the relative signal ratio values for each of the genes were retrieved using Genevestigator and the log_2_ transformed fold change values were calculated as done previously [29]. The ids of experiments used to retrieve the above values are AT00391, AT00553, AT00579, AT00575, AT00106, and AT00309 for biotic stresses in Arabidopsis; AT00560, AT00221, AT00120, and AT00645 for abiotic stresses in Arabidopsis; Os00070, Os00095, Os00082, Os00084, Os00073, and Os00011 for biotic stresses in rice; OS00008 and OS00024 for abiotic stresses in rice. Heat maps were generated with Multi Experiment Viewer software [78] (http://www.tm4.org/mev.html) with average linkage hierarchical clustering either using Euclidean or Pearson correlation as the distance metric, as the case may be. Statistical significance was tested using two-tailed Student’s t-test, p < 0.05. Expression datasets used for generating the heat maps have been provided as tables (Additional file 18: Table S8, Additional file 19: Table S9, Additional file 20: Table S10, Additional file 21: Table S11).

### Plant material and various abiotic stress treatments

Seeds of *Oryza sativa* L. genotypes IR64 and Pokkali were surface-sterilized with Bavistin (1%) and were germinated in a hydroponic system. Seedlings were grown under control conditions in Yoshida medium [79], at 28 ± 2°C and 16 h/8 h photoperiod for 12 days. Thereafter, these seedlings were subjected to different treatments for 4 hours as described previously (with some modifications) [29]. For salinity and oxidative stresses, the seedlings were shifted to Yoshida medium supplemented with 200 mM NaCl, and 10 μM methyl viologen, respectively; for drought stress, the seedlings were air-dried for four hours; for heat stress, the seedlings were shifted to a temperature of 42°C maintained in a growth chamber. Besides, to study the expression pattern in response to exogenous Abscisic acid (ABA), seedlings were shifted to Yoshida medium containing 100 μM ABA. Seedlings grown in Yoshida medium served as control.

### Expression analysis by qRT-PCR

qRT-PCR was carried out as described earlier, with some modifications [29,31]. RNA was isolated using TRIzol reagent (Life Technologies, USA) from shoot tissues of treated and untreated seedlings following the manufacturer’s protocol. The quality and integrity of the isolated RNA samples was checked by spectrophotometry and agarose gel electrophoresis under denaturing conditions. First strand cDNA was synthesized from 5 μg of total RNA (DNaseI-treated) using RevertAid™ RNase H minus cDNA synthesis kit (Thermo Fisher Scientific, USA). Primers for qRT-PCR were designed from the 3’UTR region of each of the eight genes (Additional file 22: Table S12) using Primer Express Software v3.0 (Applied Biosystems, USA). Primer-BLAST (http://www.ncbi.nlm.nih.gov/tools/primer-blast/) was used to check the specificity of the amplification from these primers. The real-time PCR mixture comprised 5 μl of cDNA (10 times diluted), 10 μl of 2× SYBR Green PCR Master Mix (Life Technologies, USA) and 100 nM of each primer (as provided in Additional file 22: Table S12) in a final volume of 20 μl. qRT-PCR was performed employing 7500™ Real-Time PCR System and software (Applied Biosystems, USA). The reaction conditions were 95°C (10 min), and 40 cycles of 15 s at 95°C and 1 min at 60°C. Melt curve analyses and agarose gel electrophoresis were carried out to ensure the specificity of the amplifications. Relative expression of each of the genes was calculated using comparative C_T_ value method [80], using *eEF-1α* as the internal control [81]. Experiments were repeated thrice (three biological replicates). Statistical significance was tested using two-tailed Student’s t-test, p < 0.05.

### Availability of supporting data

The data sets supporting the results of this article are included within the article and its additional files as cited at the relevant places in the article. Besides, the alignment matrices and the corresponding phylogenetic trees generated for the study have been submitted to TreeBASE repository (http://treebase.org/treebase-web/home.html) and are publicly accessible at http://purl.org/phylo/treebase/phylows/study/TB2:S16883.

## Additional files

Additional file 1: Table S1. Number of genes encoding histone chaperones belonging to eight different families in human, yeast, Arabidopsis, and rice. Additional file 2: Table S2. Histone chaperones from human and yeast. Additional file 3: Table S3. Putative histone chaperones from lower plants viz. *Chlamydomonas reinhardtii* (a green alga)*, Physcomitrella patens* (a bryophyte)*, Selaginella moellendorffii* (a pteridophyte), and *Picea abies* (a gymnosperm). Additional file 4: Figure S1. Multiple sequence alignment of proteins belonging to NAP family of histone chaperones from diverse eukaryotes. Additional file 5: Figure S2. Multiple sequence alignment of proteins belonging to CAF1A sub-family of histone chaperones from diverse eukaryotes. Additional file 6: Figure S3. Multiple sequence alignment of proteins belonging to CAF1B sub-family of histone chaperones from diverse eukaryotes. Additional file 7: Figure S4. Multiple sequence alignment of proteins belonging to CAF1C sub-family of histone chaperones from diverse eukaryotes. Additional file 8: Figure S5. Multiple sequence alignment of proteins belonging to HIRA family of histone chaperones from diverse eukaryotes. Additional file 9: Figure S6. Multiple sequence alignment of proteins belonging to ASF1/CIA family of histone chaperones from diverse eukaryotes. Additional file 10: Figure S7. Multiple sequence alignment of proteins belonging to NASP family of histone chaperones from diverse eukaryotes. Additional file 11: Figure S8. Multiple sequence alignment of proteins belonging to SPT6 family of histone chaperones from diverse eukaryotes. Additional file 12: Figure S9. Multiple sequence alignment of proteins belonging to SSRP sub-family of histone chaperones from diverse eukaryotes. Additional file 13: Figure S10. Multiple sequence alignment of proteins belonging to SPT16 sub-family of histone chaperones from diverse eukaryotes. Additional file 14: Table S4. Microarray-based expression values (log_2_ signal intensity) retrieved using Genevestigator for histone chaperones from Arabidopsis across different developmental stages. Additional file 15: Table S5. Microarray-based expression values (log_2_ signal intensity) retrieved using Genevestigator for histone chaperones from rice across different developmental stages. Additional file 16: Table S6. Microarray-based expression values (log_2_ signal intensity) retrieved using Genevestigator for histone chaperones from Arabidopsis across different tissues. Additional file 17: Table S7. Microarray-based expression values (log_2_ signal intensity) retrieved using Genevestigator for histone chaperones from rice across different tissues. Additional file 18: Table S8. Microarray-based expression values (log_2_ signal intensity) retrieved using Genevestigator for histone chaperones from Arabidopsis under different biotic stress conditions. Additional file 19: Table S9. Microarray-based expression values (log_2_ signal intensity) retrieved using Genevestigator for histone chaperones from rice under different biotic stress conditions. Additional file 20: Table S10. Microarray-based expression values (log_2_ signal intensity) retrieved using Genevestigator for histone chaperones from Arabidopsis under different abiotic stress conditions. Additional file 21: Table S11. Microarray-based expression values (log_2_ signal intensity) retrieved using Genevestigator for histone chaperones from rice under different abiotic stress conditions. Additional file 22: Table S12. Sequences of primers used for qRT-PCR.
