# Supplementary material for: Histone chaperones in Arabidopsis and rice: genome-wide identification, phylogeny, architecture and transcriptional regulation
Source: BMC Plant Biol. 2015 Feb 12;15:42. doi: 10.1186/s12870-015-0414-8 (PMC4357127; doi:10.1186/s12870-015-0414-8)
Supplement: Additional file 1: Table S1. — Number of genes encoding histone chaperones belonging to eight different families in human, yeast, Arabidopsis, and rice. [file 12870_2015_414_MOESM1_ESM.docx]

Additional file 1: Table S1

**Number of genes encoding histone chaperones belonging to eight different families in human, yeast, Arabidopsis, and rice.**

| **Histone chaperone family** | **NPM** | **NAP** | **CAF1** | | | **CIA/ASF1** | **HIRA** | **FACT** | | **NASP (N1/N2)** | **SPT6** |
| --- | --- | --- | --- | --- | --- | --- | --- | --- | --- | --- | --- |
| **Subunit family (Subfamily)** |  |  | **CAF1A** | **CAF1B** | **CAF1C** |  |  | **SSRP1/POB3** | **SPT16** |  |  |
| ***Homo sapiens*** | **1** | **7** | **1** | **1** | **1** | **2** | **1** | **1** | **1** | **1** | **1** |
| ***Saccharomyces cerevisiae*** | **0** | **1** | **1** | **1** | **1** | **1** | **2** | **1** | **1** | **1** | **1** |
| ***Arabidopsis thaliana*** | **0** | **6** | **1** | **1** | **6** | **2** | **1** | **1** | **1** | **1** | **2** |
| ***Oryza sativa*** | **0** | **7** | **2** | **1** | **5** | **2** | **1** | **2** | **3** | **1** | **1** |
