# Supplementary material for: Histone chaperones in Arabidopsis and rice: genome-wide identification, phylogeny, architecture and transcriptional regulation
Source: BMC Plant Biol. 2015 Feb 12;15:42. doi: 10.1186/s12870-015-0414-8 (PMC4357127; doi:10.1186/s12870-015-0414-8)
Supplement: Additional file 2: Table S2. — Histone chaperones from human and yeast. [file 12870_2015_414_MOESM2_ESM.pdf]

Additional file 2: Table S2. **Histone chaperones from human and yeast.** The table shows the nomenclature used for phylogenetic analysis along with the Uniprot id of each of the proteins.

Family/Subfamily	Nomenclature	Uniprot id	Uniprot entry name	Protein name
***Homo sapiens* (Human)**				
**NAP**	HsNAPL4	Q99733	NP1L4_HUMAN	Nucleosome assembly protein 1-like 4
	HsNAPL1	P55209	NP1L1_HUMAN	Nucleosome assembly protein 1-like 1
	HsNAPL3	Q99457	NP1L3_HUMAN	Nucleosome assembly protein 1-like 3
	HsNAPL6	A6NFF2	NP1L6_HUMAN	Putative nucleosome assembly protein 1-like 6
	HsNAPL5	Q96NT1	NP1L5_HUMAN	Nucleosome assembly protein 1-like 5
	HsNAPL2	Q9ULW6	NP1L2_HUMAN	Nucleosome assembly protein 1-like 2
	HsSET	Q01105	SET_HUMAN	Protein SET
**CAF1A**	HsCAF1A	Q13111	CAF1A_HUMAN	Chromatin assembly factor 1 subunit A
**CAF1B**	HsCAF1B	Q13112	CAF1B_HUMAN	Chromatin assembly factor 1 subunit B
**CAF1C**	HsCAF1C	Q09028	RBBP4_HUMAN	Histone-binding protein RBBP4
**CIA/ASF1**	HsCIA-II	Q9Y294	ASF1A_HUMAN	Histone chaperone ASF1A
	HsCIA-II	Q9NVP2	ASF1B_HUMAN	Histone chaperone ASF1B
**HIRA**	HsHIRA	P54198	HIRA_HUMAN	Protein HIRA
**FACT**	HsSSRP	Q08945	SSRP1_HUMAN	FACT complex subunit SSRP1
	HsSPT16	Q9Y5B9	SP16H_HUMAN	FACT complex subunit SPT16
**NASP**	HsNASP	P49321	NASP_HUMAN	Nuclear autoantigenic sperm protein
**SPT6**	HsSPT6	Q7KZ85	SPT6H_HUMAN	Transcription elongation factor SPT6
**NPM**	HsNPM	P06748	NPM_HUMAN	Nucleophosmin
***S. cerevisiae* (Yeast)**				
**NAP**	ScNAP1	P25293	NAP1_YEAST	Nucleosome assembly protein
**CAF1A**	ScCAF1p90	Q12495	RLF2_YEAST	Chromatin assembly factor 1 subunit p90
**CAF1B**	ScCAF1p60	Q04199	CAC2_YEAST	Chromatin assembly factor 1 subunit p60
**CAF1C**	ScCAF1p50	P13712	MSI1_YEAST	Chromatin assembly factor 1 subunit p50
**ASF1**	ScASF1	P32447	ASF1_YEAST	Histone chaperone ASF1
**HIRA**	ScHIR1	P32479	HIR1_YEAST	Protein HIR1
	ScHIR2	P32480	HIR2_YEAST	Protein HIR2
**FACT**	ScSSRP	Q04636	POB3_YEAST	FACT complex subunit POB3
	ScSPT16	P32558	SPT16_YEAST	FACT complex subunit SPT16
**NASP**	ScHif1	Q12373	HIF1_YEAST	HAT1-interacting factor 1
**SPT6**	ScSPT6	P23615	SPT6_YEAST	Transcription elongation factor SPT6
