# Supplementary material for: Histone chaperones in Arabidopsis and rice: genome-wide identification, phylogeny, architecture and transcriptional regulation
Source: BMC Plant Biol. 2015 Feb 12;15:42. doi: 10.1186/s12870-015-0414-8 (PMC4357127; doi:10.1186/s12870-015-0414-8)
Supplement: Additional file 3: Table S3. — Putative histone chaperones from lower plants viz. Chlamydomonas reinhardtii (a green alga), Physcomitrella patens (a bryophyte), Selaginella moellendorffii (a pteridophyte), and Picea abies (a gymnosperm). [file 12870_2015_414_MOESM3_ESM.docx]

**Additional file 3: Table S3**

Additional file 3: Table S3. **Putative histone chaperones from lower plants viz. *Chlamydomonas reinhardtii* (a green alga)*, Physcomitrella patens* (a bryophyte)*, Selaginella moellendorffii* (a pteridophyte), and *Picea abies* (a gymnosperm). For phylogenetic reconstruction (Figure 3, 4 and 5) the locus id of genes encoding putative histone chaperone from *S. moellendorffii* and *P. abies* were purposefully prefixed with ‘Sm’ and ‘Pa’, respectively, to represent the name of the species.**

| Family/Subfamily | *Chlamydomonas reinhardtii* | *Physcomitrella patens* | *Selaginella moellendorffii* | *Picea abies* |
| --- | --- | --- | --- | --- |
|  | **Locus id** | **Locus id** | **Locus id** | **Locus id** |
| NAP | Cre09.g416350.t1.2 | Phpat.003G023500.2.p | 74970 | MA_10427220g0020 |
|  | Cre02.g073550.t1.2 | Phpat.025G017300.2.p | 443393 | MA_25056g0010 |
|  | Cre02.g095082.t1.1 | Phpat.025G017300.3.p | 89449 | MA_457513g0010 |
|  | Cre06.g307600.t1.2 | Phpat.025G017300.1.p |  | MA_74956g0010 |
|  |  | Phpat.018G053500.1.p |  |  |
|  |  | Phpat.018G053500.2.p |  |  |
|  |  | Phpat.018G053500.3.p |  |  |
|  |  | Phpat.018G053800.1.p |  |  |
|  |  | Phpat.006G042300.1.p |  |  |
| CAF1A | Cre12.g550152.t1.1 | Phpat.003G098500.1.p | 405999 | MA_27625g0020 |
| CAF1B | Cre13.g575950.t1.2 | Phpat.001G056500.1.p | 109484 | MA_6422g0020 |
|  |  | Phpat.001G056500.2.p | 267866 |  |
| CAF1C | Cre12.g523200.t1.1 | Phpat.022G022400.1.p | 233646 | MA_142023g0010 |
|  | Cre17.g724850.t1.2 | Phpat.015G071000.1.p | 107569 | MA_10425957g0010 |
|  |  | Phpat.015G071000.2.p | 121554 | MA_142023g0010 |
|  |  | Phpat.015G071000.3.p | 134213 | MA_10425957g0010 |
|  |  | Phpat.012G061300.1.p | 431142 |  |
|  |  | Phpat.012G061300.2.p |  |  |
| ASF1 | Cre08.g376350.t1.2 | Phpat.008G092000.1.p | 90000 | MA_16730g0010 |
|  | Cre08.g376350.t2.1 | Phpat.023G080100.1.p |  | MA_73031g0010 |
|  |  | Phpat.023G079900.1.p |  | MA_10428471g0010 |
|  |  | Phpat.023G079900.2.p |  | MA_10428471g0020 |
|  |  | Phpat.023G079900.3.p |  |  |
|  |  | Phpat.023G079900.4.p |  |  |
|  |  | Phpat.023G079900.5.p |  |  |
|  |  | Phpat.024G076800.1.p |  |  |
| HIRA | Cre13.g575950.t1.2 | Phpat.027G005500.2.p | 109484 | MA_44386g0010 |
|  |  | Phpat.027G005500.1.p | 267866 |  |
|  |  | Phpat.016G064900.1.p | 94452 |  |
| NASP |  | Phpat.024G019400.2.p | 418586 | MA_102821g0020 |
|  |  | Phpat.024G019400.1.p |  |  |
| SPT6 | Cre09.g403182.t1.1 | Phpat.005G088200.2.p | 164675 | MA_13648g0020 |
|  |  | Phpat.005G088200.3.p |  |  |
|  |  | Phpat.005G088200.4.p |  |  |
|  |  | Phpat.005G088200.1.p |  |  |
|  |  | Phpat.005G089600.1.p |  |  |
| SSRP | Cre01.g029450.t1.1 | Phpat.009G018700.1.p | 166865 |  |
| SPT16 | Cre12.g540076.t1.1 | Phpat.013G054600.1.p | 170207 | MA_10430230g0030 |
|  |  |  |  | MA_10427450g0010 |
