# Supplementary figures and images for: Histone chaperones in Arabidopsis and rice: genome-wide identification, phylogeny, architecture and transcriptional regulation
Source: BMC Plant Biol. 2015 Feb 12;15:42. doi: 10.1186/s12870-015-0414-8 (PMC4357127; doi:10.1186/s12870-015-0414-8)

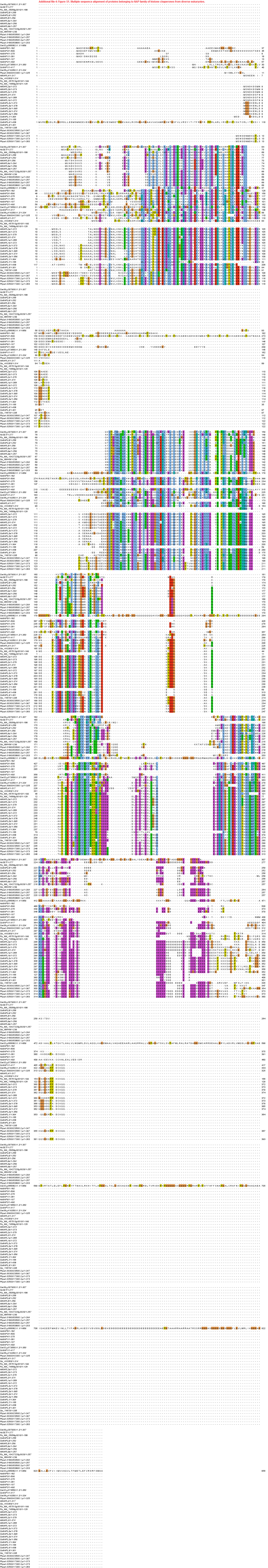

Supplement: Additional file 4: Figure S1. — Multiple sequence alignment of proteins belonging to NAP family of histone chaperones from diverse eukaryotes. [file 12870_2015_414_MOESM4_ESM.png]

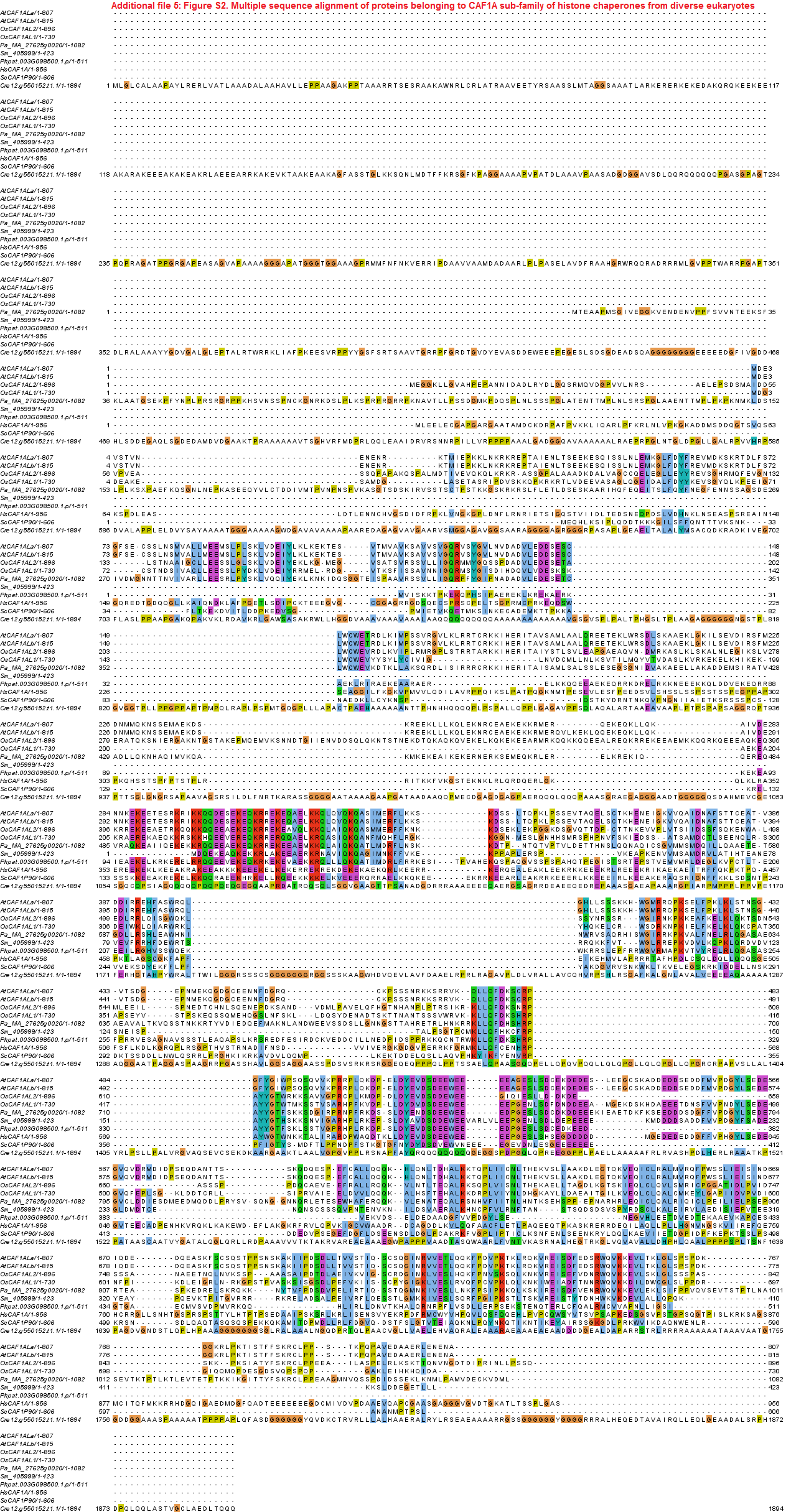

Supplement: Additional file 5: Figure S2. — Multiple sequence alignment of proteins belonging to CAF1A sub-family of histone chaperones from diverse eukaryotes. [file 12870_2015_414_MOESM5_ESM.png]

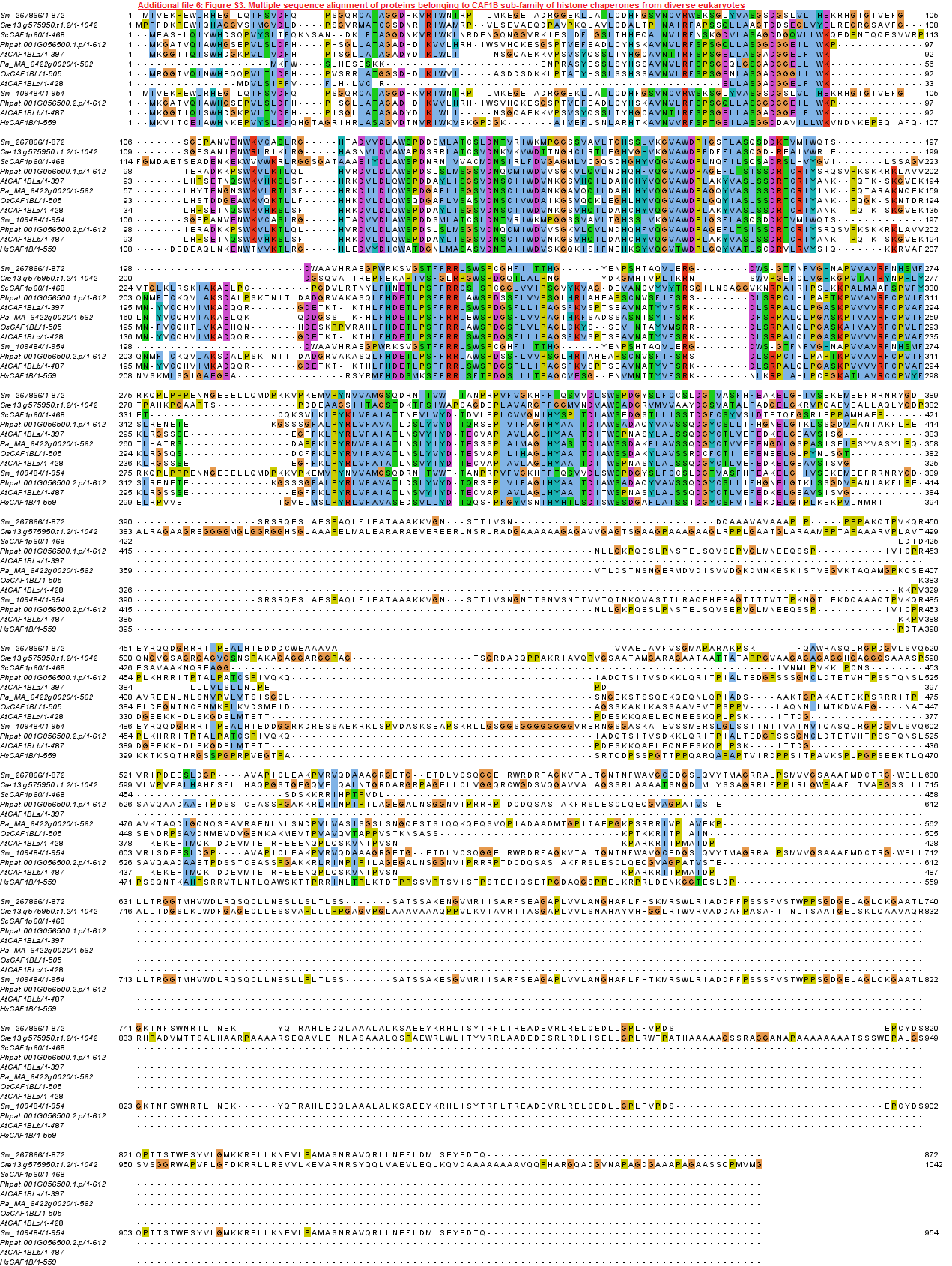

Supplement: Additional file 6: Figure S3. — Multiple sequence alignment of proteins belonging to CAF1B sub-family of histone chaperones from diverse eukaryotes. [file 12870_2015_414_MOESM6_ESM.png]

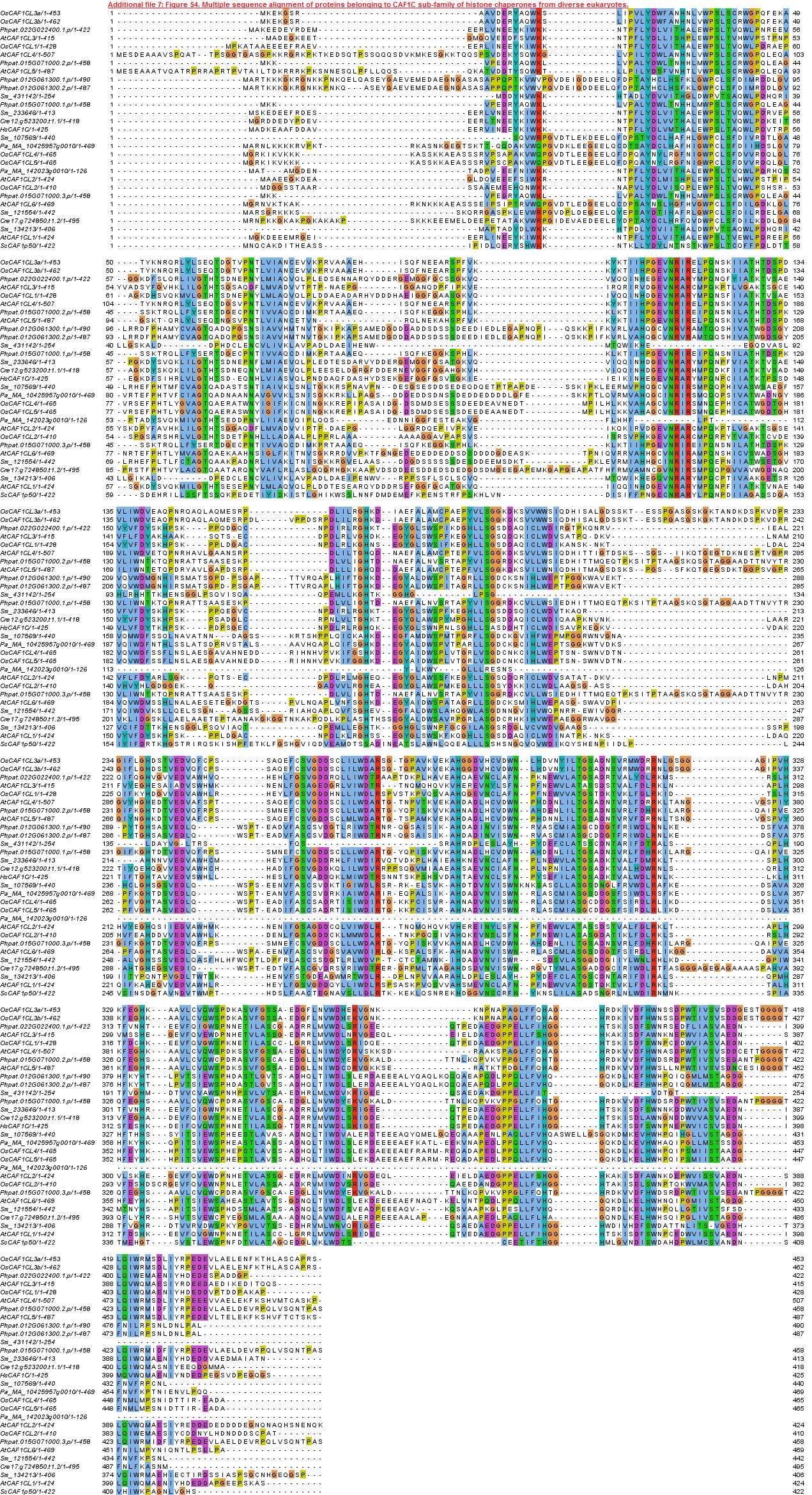

Supplement: Additional file 7: Figure S4. — Multiple sequence alignment of proteins belonging to CAF1C sub-family of histone chaperones from diverse eukaryotes. [file 12870_2015_414_MOESM7_ESM.png]

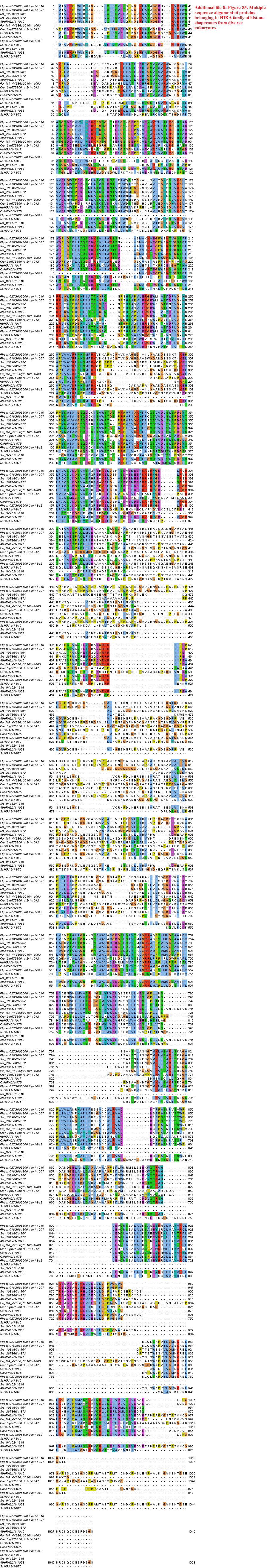

Supplement: Additional file 8: Figure S5. — Multiple sequence alignment of proteins belonging to HIRA family of histone chaperones from diverse eukaryotes. [file 12870_2015_414_MOESM8_ESM.png]

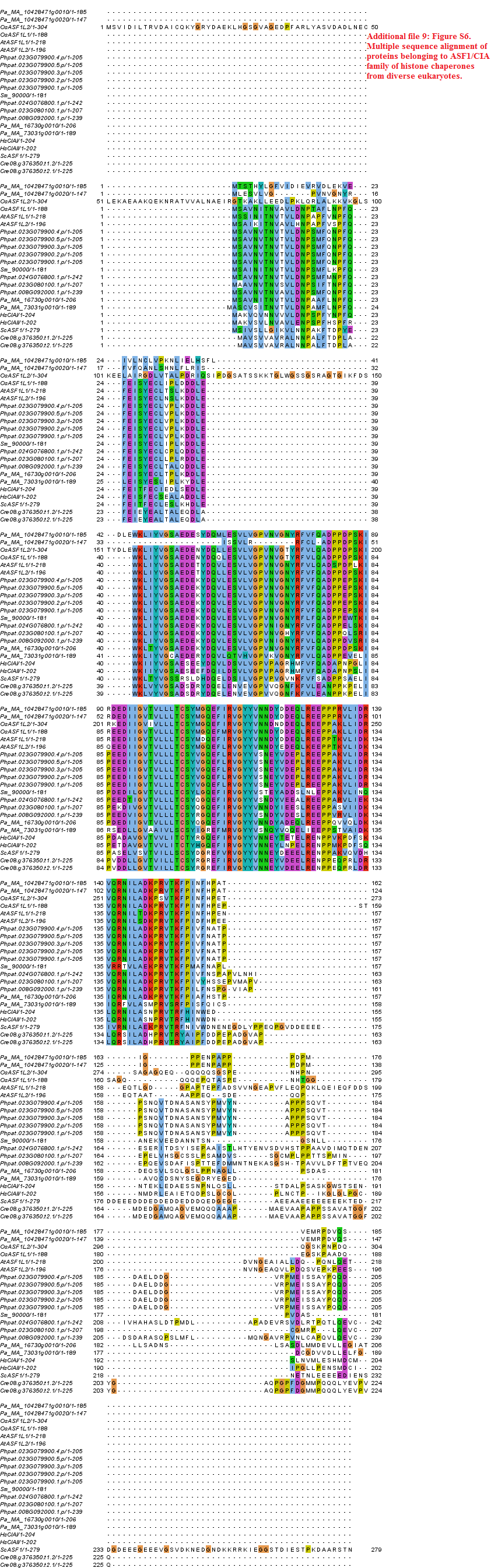

Supplement: Additional file 9: Figure S6. — Multiple sequence alignment of proteins belonging to ASF1/CIA family of histone chaperones from diverse eukaryotes. [file 12870_2015_414_MOESM9_ESM.png]

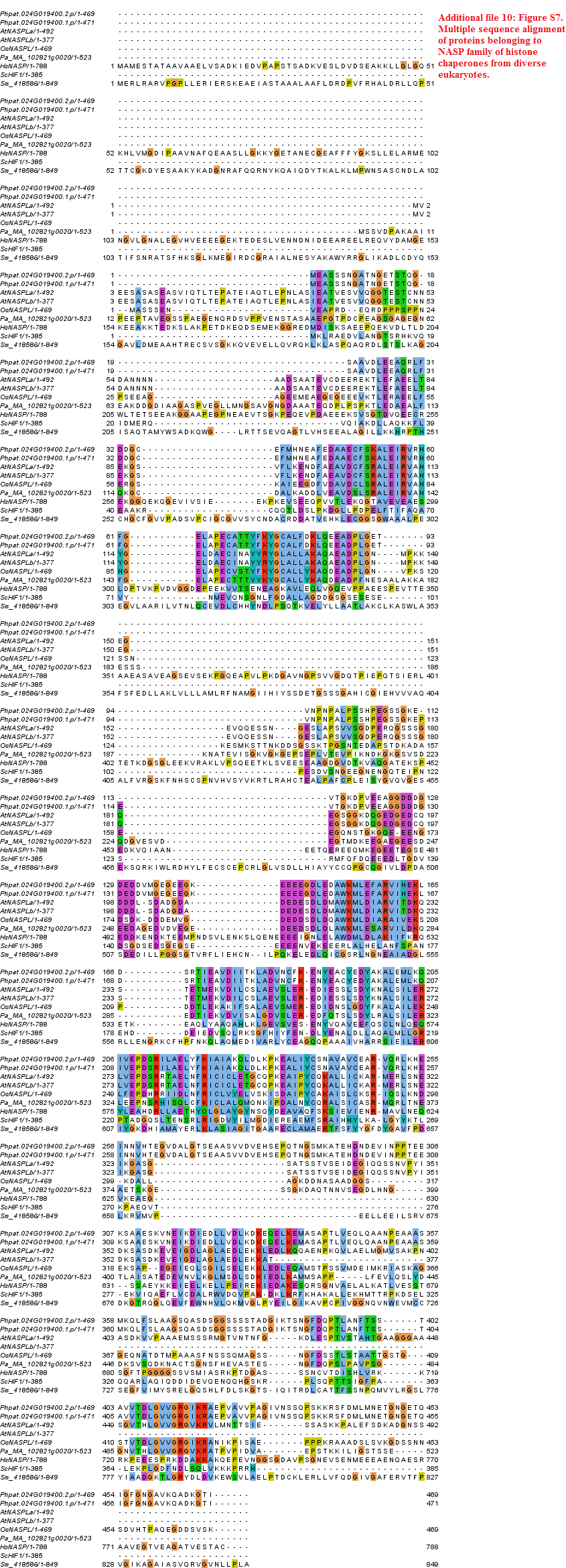

Supplement: Additional file 10: Figure S7. — Multiple sequence alignment of proteins belonging to NASP family of histone chaperones from diverse eukaryotes. [file 12870_2015_414_MOESM10_ESM.png]

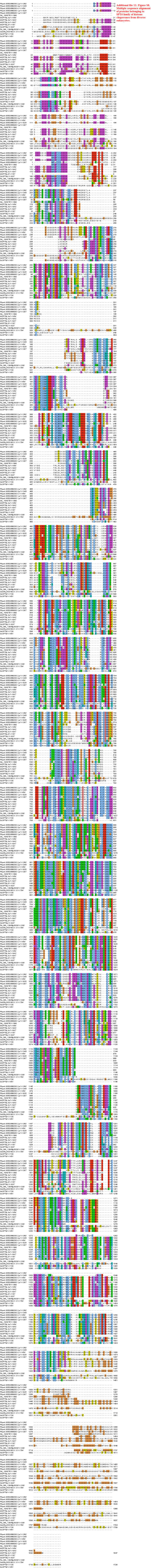

Supplement: Additional file 11: Figure S8. — Multiple sequence alignment of proteins belonging to SPT6 family of histone chaperones from diverse eukaryotes. [file 12870_2015_414_MOESM11_ESM.png]

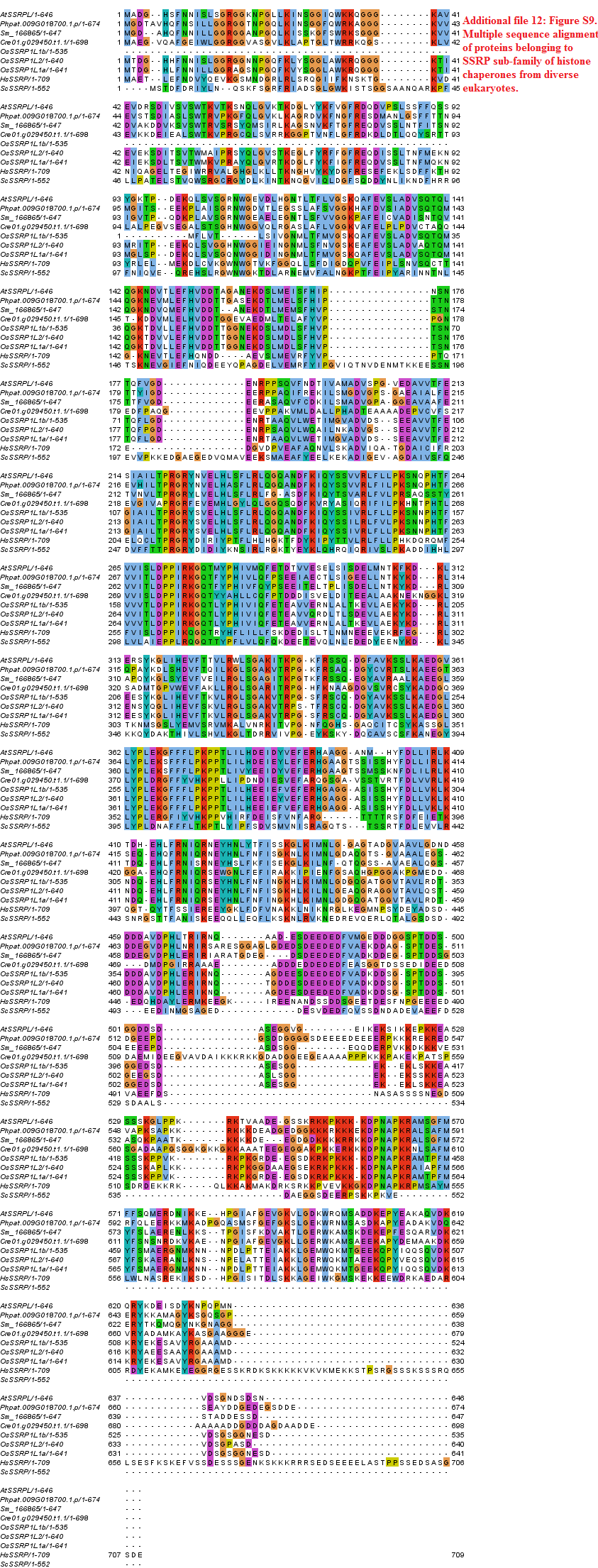

Supplement: Additional file 12: Figure S9. — Multiple sequence alignment of proteins belonging to SSRP sub-family of histone chaperones from diverse eukaryotes. [file 12870_2015_414_MOESM12_ESM.png]

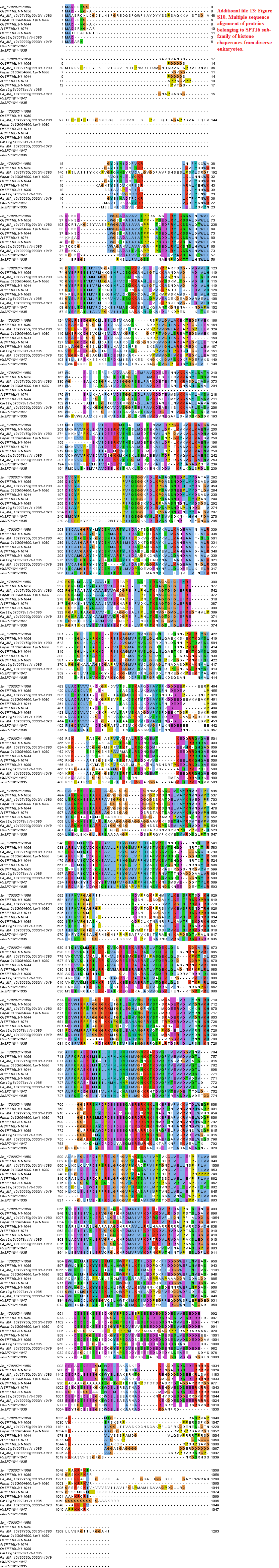

Supplement: Additional file 13: Figure S10. — Multiple sequence alignment of proteins belonging to SPT16 sub-family of histone chaperones from diverse eukaryotes. [file 12870_2015_414_MOESM13_ESM.png]
