# Supplementary material for: Histone chaperones in Arabidopsis and rice: genome-wide identification, phylogeny, architecture and transcriptional regulation
Source: BMC Plant Biol. 2015 Feb 12;15:42. doi: 10.1186/s12870-015-0414-8 (PMC4357127; doi:10.1186/s12870-015-0414-8)
Supplement: Additional file 22: Table S12. — Sequences of primers used for qRT-PCR. [file 12870_2015_414_MOESM22_ESM.pdf]

Additional file 22: Table S12. **Sequences of primers used for qRT-PCR.**

**Gene**	**Primer name**	**Primer Sequence (5'-3')**
OsCAF1AL2	OsCAF1AL2 Fw	GGTTGAGGAGCAGCAGCATA
	OsCAF1AL2 Re	TGCGACCGAAGAGTCCATTC
OsNAPL5	OsNAPL5 Fw	CTGGACAACTTATGTCTGCAGAAAG
	OsNAPL5 Re	CAGCTAAGTCGAGCACCACAAC
OsNAPL6	OsNAPL6 Fw	GCCAGTCTGATGGGCTGTTATT
	OsNAPL6 Re	GCACGGTTTCGTAAACAATGATC
OsCAF1CL5	OsCAF1CL5 Fw	GATGTCGCCCGAATGAGGAT
	OsCAF1CL5 Re	GGCAAAAATATACAAACGATCGAGG
OsCAF1CL2	OsCAF1CL2Fw	CGTTTTCGTTTGACACCTCCA
	OsCAF1CL2 Re	AGCAACCAGGCCAAAGATCA
OsSPT16L2	OsSPT16L2 Fw	CCTGTCGTTAGTTTCGGTGGT
	OsSPT16L2 Re	ACCATGTGCAGTAAAACGAGA
OsSSRPL2	OsSSRPL2Fw	GACAGGATACGGCTAGTGGC
	OsSSRPL2 Re	ACACTCATAGAACGGGACTCG
OsCAF1BL	OsCAF1BLFw	AAGGGGTATGATGGCTCCCT
	OsCAF1BL Re	ATGTCCACAAGGCGACTGAG
eEF-1α (endogenous control)	eEF-1α Fw	TTTCACTCTTGGTGTGAAGCAGAT
	eEF-1α Re	GACTTCCTTCACGATTTCATCGTAA
